# Peri‐Xanthenoxanthene‐Based Covalent Organic Frameworks for High‐Performance Aqueous Zn‐Ion Hybrid Supercapacitors

**DOI:** 10.1002/smsc.202400031

**Published:** 2024-04-26

**Authors:** Cataldo Valentini, Verónica Montes‐García, Luca Cusin, Dawid Pakulski, Mateusz Wlazło, Paolo Samorì, Artur Ciesielski

**Affiliations:** ^1^ Nanochemistry LaboratoryInstitut de Science et d’Ingénierie Supramoléculaires (I.S.I.S.) Université de Strasbourg & CNRS 8 allée Gaspard Monge 67000 Strasbourg France; ^2^ Centre for Advanced Technologies Adam Mickiewicz University Uniwersytetu Poznańskiego 10 61‐614 Poznań Poland; ^3^ Chemical and Biological Systems Simulation Lab Centre of New Technologies University of Warsaw 02‐097 Warsaw Poland

**Keywords:** covalent organic frameworks, polyconjugated molecules, Zn‐ion hybrid supercapacitors

## Abstract

Aqueous zinc‐ion hybrid supercapacitors (Zn‐HSCs) are promising devices for sustainable and efficient energy storage. However, they suffer from a limited energy density compared to lithium‐ion batteries. This limitation can be overcome by developing novel electrode materials, with covalent organic frameworks (COFs) standing out as a particularly intriguing option. Herein, peri‐xanthenoxanthene (PXX) has been integrated for the first time into a COF scaffold to take advantage of its straightforward synthesis, chemical stability, π‐conjugated backbone, and heteroatom content endowing reversible redox reactions at low potentials. Two novel hexagonal COFs have been designed and synthesized by tethering of a PXX‐diamine unit having a C_2_ symmetry with two distinct tris‐aldehydes acting as C_3_‐symmetric cornerstones, i.e., triformyl benzene (TFB) and triformylphloroglucinol (Tp), ultimately yielding COF PXX(PhNH_2_)_2_‐TFB and COF PXX(PhNH_2_)_2_‐Tp, respectively. As cathodes in Zn‐HSCs, COF PXX(PhNH_2_)_2_‐Tp exhibits a remarkable specific capacitance, energy, and power densities (237 F g^−1^, 106.6 Wh kg^−1^, and 3.0 kW kg^−1^, respectively), surpassing those of COF PXX(PhNH_2_)_2_‐TFB (109 F g^−1^, 49.1 Wh kg^−1^, and 0.67 kW kg^−1^). Importantly, both COFs display outstanding long‐term stability, over 5000 charge/discharge cycles, with capacitance retention >92%. These findings underscore the potential of PXX‐based COFs as high‐performance cathode materials for HSCs, thereby offering a promising new avenue for energy storage technologies.

## Graphical abstract

Herein, two unprecedentedly reported hexagonal covalent organic frameworks based on peri‐xanthenoxanthene are synthetized. Their performance as cathodes in zinc‐ion hybrid supercapacitors (Zn‐HSCs) is assessed, obtaining competitive performance (capacitance of 237 F g^−1^ at 10 mV s^−1^ and 92% capacitance retention after 5000 charge/discharge cycles) compared to the other reported cathode materials for Zn‐HSCs.

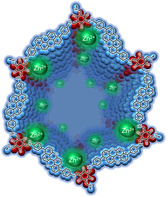

## 1 Introduction

Covalent organic frameworks (COFs) represent an emerging class of nanostructured crystalline polymers characterized by a porous and well‐defined 2D or 3D structure.^[^ ^1^, ^2^, ^3^ ^]^ Highly ordered COFs can be formed by exploiting reversible chemical bonds to connect conformationally rigid building blocks by mastering dynamic covalent chemistry (DCC) strategies.^[^ ^4^ ^]^ Among the DCC reactions employed for the synthesis of COFs, the most popular ones are boronic acid trimerization, boronic acid and catechol condensation, imine condensation, hydrazone formation, azine formation, and imide formation.^[^ ^2^, ^5^, ^6^, ^7^, ^8^, ^9^ ^]^ The great variety of synthetic protocols coupled with the remarkably high surface areas of these low‐dimensional porous materials positions COFs as well suited for numerous applications, including catalysis, gas and liquid purification, and energy storage.^[^ ^10^, ^11^, ^12^, ^13^, ^14^, ^15^ ^]^

The first example of COF serving as an electrode material dates back just a decade, when, thanks to its remarkable chemical stability, β‐ketoenamine‐linked COF was exploited as supercapacitor electrode material.^[^ ^16^ ^]^ Following such a milestone, numerous examples have been reported, aiming to attain optimal performance in diverse energy storage applications.^[^ ^15^, ^17^ ^]^ Among the portfolio of technologies available, rechargeable aqueous zinc‐ion hybrid supercapacitors (Zn‐HSCs) are considered being particularly promising because the use of zinc metal as the anode^[^ ^18^ ^]^ has several advantages, including the abundance of zinc, its chemical stability, relatively low cost (estimated at 3 $ kg^−1^ in 2022), high theoretical capacity (820 mAh g^−1^), and low redox potential (–0.763 V vs standard H_2_ electrode).^[^ ^18^ ^]^ The major limitation associated with current Zn‐HSCs lies in the comparatively lower energy densities that have been achieved, especially when compared with lithium‐ion batteries (LIBs). Strategies to enhance energy density focus on increasing either capacitance/capacity or the operating voltage window. While expanding the voltage window involves the exploration of new electrolytes like ionic liquids, enhancing capacitance or capacity can be achieved through innovative electrode material designs with multielectron redox chemistry.^[^ ^19^, ^20^ ^]^ Surface functionalization methods, such as coating or doping, can alter electrode properties to boost ion adsorption and surface reactions, thereby improving energy density and charge–discharge performance, reaching the levels of LIBs.

Fortunately, the utilization of COF‐based cathodes holds the potential to make substantial advancements in the field of energy storage. The structure as well as physical and chemical properties of COFs can be customized on‐demand via the ad hoc selection of the molecular building monomers and through postsynthetic functionalization methods.^[^ ^21^ ^]^ Maximizing surface area, pore size, electronic and ionic conductivity, along with enhancing electrochemical activity, has been recognized as crucial for achieving optimal electrochemical performance.^[^ ^22^ ^]^

Within the immense library of readily available COF building units, polyconjugated molecules stand out as one of the most extensively investigated monomers, mainly due to their rigid structure and extended π‐conjugated backbone, imparting electroactive properties.^[^ ^3^, ^23^, ^24^ ^]^ The incorporation of heteroatoms like nitrogen, oxygen, and sulfur in conjugated scaffolds can introduce additional active sites that facilitate reversible coordination with metal ions, like Zn^2+^, thereby yielding enhanced device performance. This was clearly demonstrated by Alshareef et al. by enriching the COF pores with nitrogen atoms, leading to superior performance (capacity 247 mAh g^−1^ at 0.1 A g^−1^) and high cyclability with only 0.38% capacity loss after 10 000 charge/discharge cycles at 1.0 A g^−1^.^[^ ^25^ ^]^

Among the reported oxygen‐doped polyconjugated molecules, peri‐xanthenoxanthene (PXX) has garnered a significant interest in the field of organic electronics and photocatalysis.^[^ ^26^, ^27^, ^28^, ^29^ ^]^ Within the PXX framework, the presence of oxygen atoms at positions 6 and 12 enhances its chemical stability through the passivation effect of oxygen. Furthermore, the inclusion of oxygen dopants alters the electronic structure and enhances the molecule's reversible oxidizability, facilitating redox reactions at lower potentials. This characteristic can be beneficial for numerous applications, especially in electrochemical systems, where the decreased oxidation potential enables more efficient and straightforward electron transfer processes. Noteworthy, Sony secured a patent for a series of organic semiconductors built upon the PXX molecule due to its outstanding performance.^[^ ^27^, ^30^ ^]^ Grätzel et al. reported the synthesis of PXX and its derivatives using cost‐effective binaphthols (BINOLs) as primary building blocks, rendering PXX intriguing as an electroactive molecule with the bonus of low production cost.^[^ ^31^ ^]^ Despite its enormous potential, PXX has never been integrated in a COF structure and PXX‐based molecules have never been reported for energy storage applications.

In this work, we engineer and synthesize for the first time PXX‐based COFs with a hexagonal topology that embeds diamino‐functionalized PXX molecules as linkers with C_2_ symmetry (PXX(PhNH_2_)_2_). Two different aldehyde‐based monomers, which differ by the presence/absence of hydroxyl groups in the 2,4,6 position in the benzene‐1,3,5‐tricarbaldehyde scaffold, are employed as C_3_‐symmetric cornerstones. In particular, we have chosen triformyl benzene (TFB) to create an imine‐linked COF (COF PXX(PhNH_2_)_2_‐TFB), and additionally, we have utilized triformylphloroglucinol (Tp) to obtain a keto‐enamine‐linked COF (COF PXX(PhNH_2_)_2_‐Tp). This comparative analysis between the two COFs is aimed at elucidating the impact of the selected cornerstone and bonding type on the materials’ structural characteristics and their electrochemical performance. We utilized both a three‐electrode system and an aqueous Zn‐HSC to evaluate the electrochemical performance of the two COF electrode materials. The improved electrochemical performance of COF PXX(PhNH_2_)_2_‐Tp as a cathode material in aqueous Zn‐HSCs is associated with several structural characteristics, including enhanced surface area, pore dimensions, and the presence of redox‐active groups, in particular when benchmarked with COF PXX(PhNH_2_)_2_‐TFB. This correlation provides valuable insights for the efficient fabrication of high‐performance aqueous Zn^2+^ ion‐based energy storage devices.

## 2 Results and Discussion

### 2.1 COFs Synthesis

**Scheme 1:**
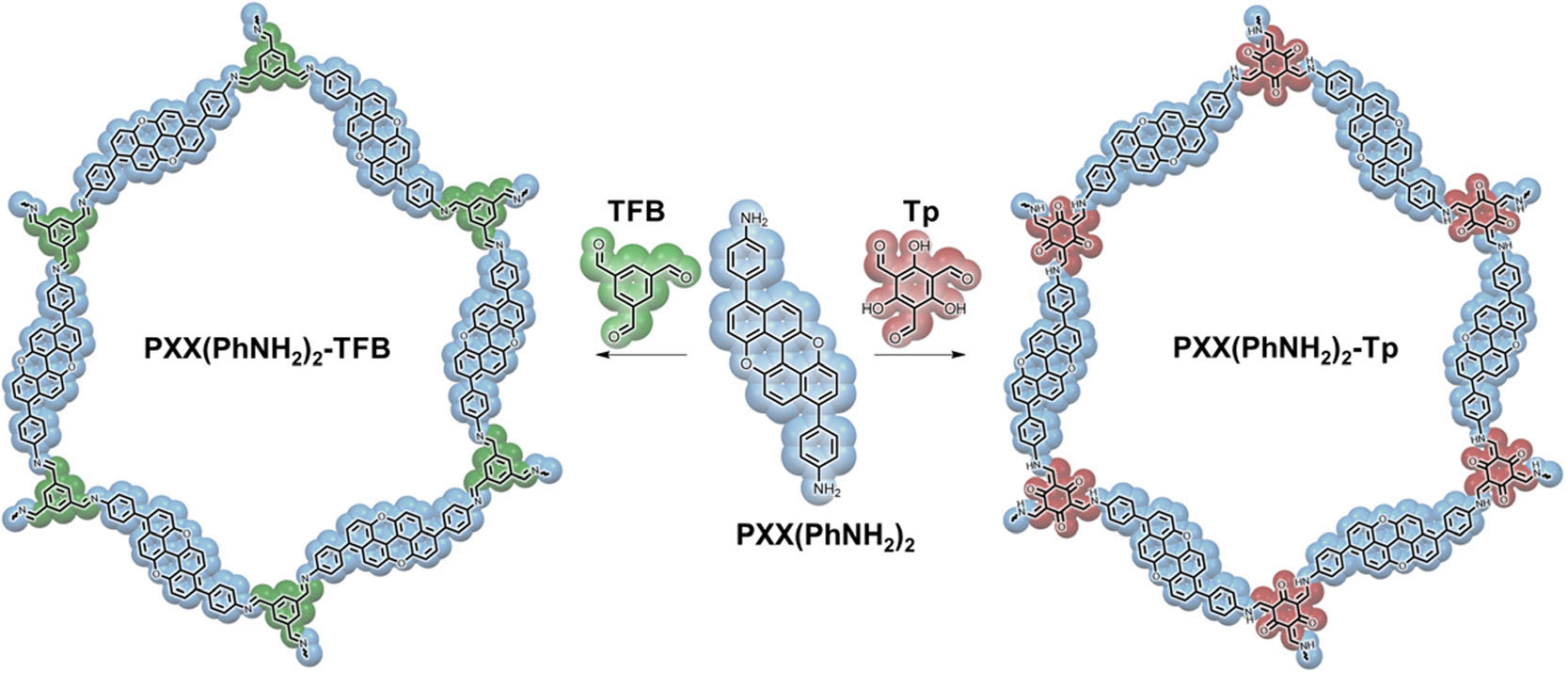
Synthetic scheme for the preparation of COF PXX(PhNH_2_)_2_‐TFB and COF PXX(PhNH_2_)_2_‐Tp.

As a molecular linker of our COFs, PXX(PhNH_2_)_2_ was selected over generic all‐carbon polyconjugated molecules for several reasons. First, the renowned electrochemical stability during oxidation and reduction reactions would increase the cyclability of Zn‐HSCs.^[^ ^26^, ^32^ ^]^ Additionally, the oxygen heteroatoms within the PXX(PhNH_2_)_2_ molecule could serve as coordination sites for Zn^2+^ ions, further boosting its electrochemical performance of Zn‐HSCs. Molecular electrostatic potential (MESP) mapping was employed to determine specific locations where Zn^2+^ ions are coordinated (Figure S4, Supporting Information). Regions with a more negative MESP, indicative of high electronegativity, were identified in the oxygen heteroatoms of the PXX(PhNH_2_)_2_ scaffold, highlighting their reactivity sites for facilitating Zn^2+^ ions uptake during the discharge process in energy storage systems. To enhance the stability of the monomer, the exploited synthetic approach yielding PXX(PhNH_2_)_2_ relies on the use of *tert*‐butyl carbonyl (Boc) protected –NH_2_ groups. The synthesis of the ligand PXX(PhNHBoc)_2_ started with the oxidative copper catalyzed O‐cyclization of BINOL, following the protocol developed by Kamei et al. (Scheme S1, Supporting Information), resulting in a 77% yield PXX.^[^ ^33^ ^]^ Subsequently, PXX was selectively brominated at positions 3 and 9 using *N*‐bromosuccinimmide in *ortho*‐dichlorobenzene at 100 °C for 4 h. PXX(PhNHBoc)_2_ was obtained through a Suzuki cross‐coupling reaction between a commercially available boronic ester and the previously prepared PXX‐Br_2_. The compound PXX(PhNHBoc)_2_ was quantitatively deprotected, yielding PXX(PhNH_2_)_2_, using a solution of dichloromethane/trifluoroacetic acid (CH_2_Cl_2_/TFA) in a 1:1 ratio, immediately before the polymerization reaction. For the synthesis of COFs, we have selected two distinct aldehyde‐based monomers: TFB and Tp. These two COFs will enable us to investigate how the type of linkage (imine vs enamine) affects the Zn‐HSC cyclability. Additionally, we aim to understand how the presence of redox‐active carbonyl groups impacts the capacitance and energy density of the Zn‐HSC. The synthesis of the COFs was performed via a solvothermal protocol using a 1:1 ratio of dioxane/mesitylene, along with 6 M acetic acid as catalyst, at 120 °C for 5 days (**Scheme** **1**). All unreacted monomers were subsequently removed through a 24 h Soxhlet purification using tetrahydrofuran.

### 2.2 Structural Characterization

The formation of COF PXX(PhNH_2_)_2_‐TFB and COF PXX(PhNH_2_)_2_‐Tp was first confirmed by attenuated total reflection Fourier‐transform infrared (ATR‐FTIR) spectroscopy (Figure S5–S7, Supporting Information). The simulated IR of Tp and TFB building units reveals the characteristic C═O stretching vibration at 1696 and 1690 cm^−1^, respectively, which are shifted toward lower wavenumbers (1619 and 1612 cm^−1^ for COF PXX(PhNH_2_)_2_‐TFB and COF PXX(PhNH_2_)_2_‐Tp, respectively) and can be ascribed to C═N stretching vibration, in full agreement with other reported imine‐linked COFs.^[^ ^16^, ^34^, ^35^ ^]^ Moreover, the IR spectrum of COF PXX(PhNH_2_)_2_‐Tp displays a signal at 1298 cm^−1^ that can be attributed to C—N enamine stretching.^[^ ^35^ ^]^ Importantly, the signals associated to the NH_2_ group (1566, 1704, and 3128–3239 cm^−1^) from PXX(PhNH_2_)_2_ building unit are absent in both COFs, providing evidence for the complete COF formation.

**Figure 1:**
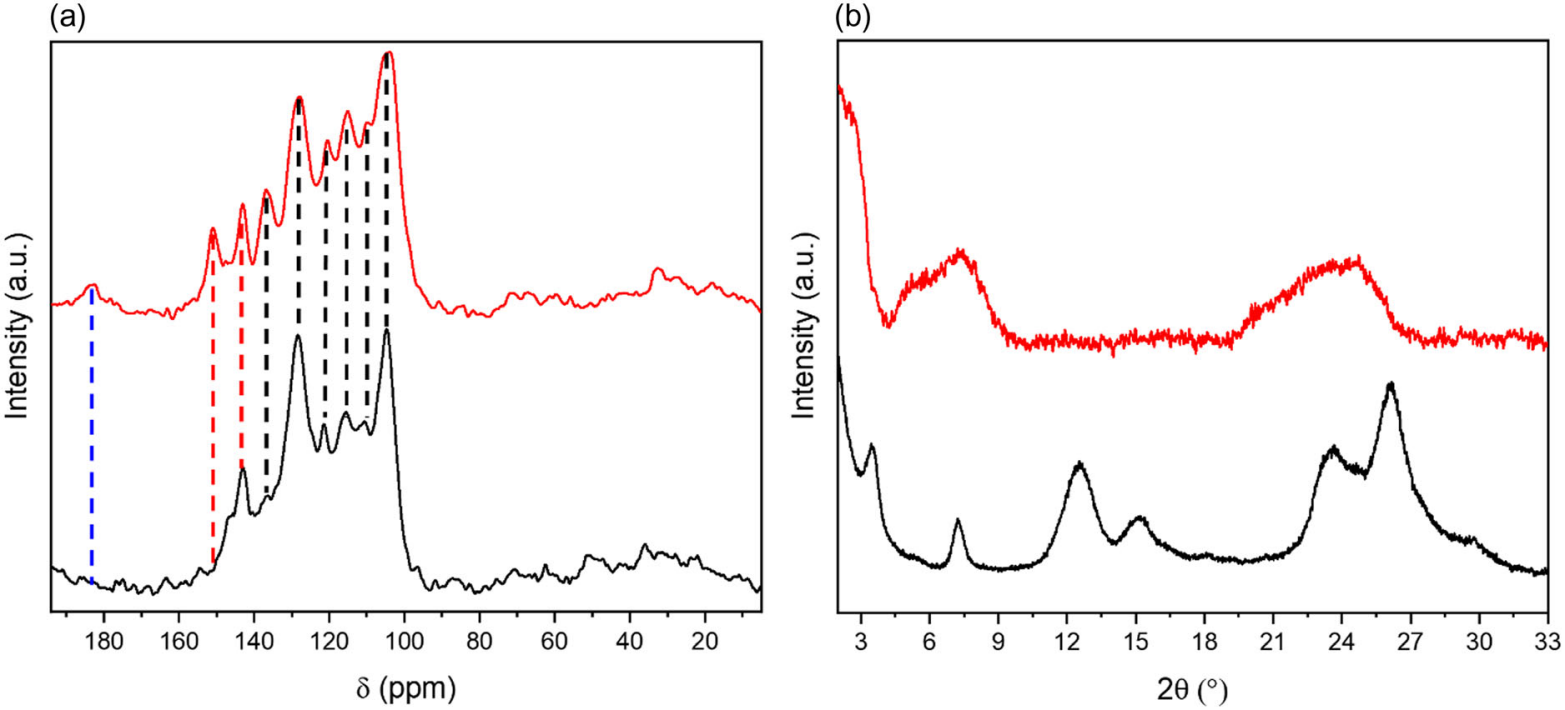
a) ^13^C ssNMR spectra and b) PXRD of PXX(PhNH_2_)_2_‐Tp (red lines) and PXX(PhNH_2_)_2_‐TFB (black lines).

To reveal the presence of imine, imine/phenol, or β‐ketoenamine bonds within the COF structures and provide additional validation of the successful synthesis, we performed cross‐polarization magic angle spinning solid‐state ^13^C nuclear magnetic resonance (NMR) analysis. The signals associated with imine and enamine linkages appear within the 140–160 ppm range.^[^ ^36^, ^37^ ^]^ COF PXX(PhNH_2_)_2_‐TFB spectrum displays a single sharp signal, confirming the exclusive presence of imine linkage. In contrast, COF PXX(PhNH_2_)_2_‐Tp spectrum exhibits two distinct signals, indicating the simultaneous existence of imine and enamine linkage (indicated by red dashed lines on **Figure** **1**a). Importantly, the ketone carbon signals of the keto‐enamine form in COF PXX(PhNH2)2‐Tp is detected at a chemical shift around 185 ppm and absent in COF PXX(PhNH_2_)_2_‐TFB (Figure 1a, marked by blue dashed line).

To further corroborate the chemical composition of both COFs, X‐ray photoelectron spectroscopy (XPS) analyses were performed. The high‐resolution C1s, N1s, and O1s spectra of COF PXX(PhNH_2_)_2_‐TFB and COF PXX(PhNH_2_)_2_‐Tp are presented in Figure S8 (Supporting Information). In the C1s spectra of both samples, apart from the dominant carbon–carbon peak at 284.7 eV (C═C), an additional peak is observed at around 287.5 eV, indicative of carbon‐heteroatom bonds (C—O and C—N). Moreover, in COF PXX(PhNH_2_)_2_‐Tp a distinctive peak attributed to carbonyl C═O bond is clearly discernible (Figure S8b, Supporting Information). Conversely, the N1s spectrum of COF PXX(PhNH_2_)_2_‐TFB (Figure S8c, Supporting Information) displays one peak at ≈400 eV, corresponding to the environment of C═N bond. Differently, the N1s spectrum of COF PXX(PhNH_2_)_2_‐Tp (Figure S8d, Supporting Information) displays two peaks at ≈400 and ≈399 eV corresponding to the chemical bonds C═N and C—N, respectively, thereby confirming the formation of imine bonds in both cases, and the enamine bond in the COF PXX(PhNH_2_)_2_‐Tp consistent with the findings from solid‐state NMR (ssNMR) and FTIR analyses. The O1s spectrum of COF PXX(PhNH_2_)_2_‐TFB (Figure S8e, Supporting Information) predominantly exhibits a peak at ≈533 eV, highlighting the presence of etheric moieties (C—O—C) within the PXX monomer structure. Noteworthy, in COF PXX(PhNH_2_)_2_‐Tp (Figure S8f, Supporting Information), an additional peak at ≈531.5 eV is evident, which can be ascribed to C═O carbonyl group.

The crystallinity of the bulk COFs was assessed by powder X‐ray diffraction (PXRD) analyses (Figure 1b). PXRD reveals distinct structural differences between the two COFs. While COF PXX(PhNH_2_)_2_‐Tp exhibits the first peak at a low angle of 2.8° (2*θ*), along with broad diffraction features at 5.2°, 7.7°, and 24.54°, COF PXX(PhNH_2_)_2_‐TFB displays a higher degree of crystallinity compared to PXX(PhNH_2_)_2_‐Tp, with sharper reflections at 3.5°, 7.2°, 12.5°, 15.2°, 23.6°, and 26.1°. To elucidate the crystal structure of the as‐prepared COFs and calculate the unit cell parameters (Table S1 and S2, Supporting Information), we conducted simulations of the powder XRD patterns using density functional theory (DFT) calculations (Figure S9–S12, Supporting Information). Two different 2D structures were built, eclipsed stacking (AA) as well as staggered stacking (AB) models (Figure S8–S9, Supporting Information). As illustrated in Figure S11 and S12 (Supporting Information), the primary distinction between the AA and AB models becomes apparent within the range of approximately 12°–15°. Notably, only the AB stacking model exhibits peaks in this region. Consequently, we can establish that COF PXX(PhNH_2_)_2_‐TFB features an AB stacking pattern, whereas COF PXX(PhNH_2_)_2_‐Tp showcases an AA stacking pattern. A calculation based on Bragg's law reveals an average interlayer distance of 3.4 Å for both COF PXX(PhNH_2_)_2_‐Tp and COF PXX(PhNH_2_)_2_‐TFB, which provide sufficient space for the transfer of hydrated Zn^2+^ ions (diameter of 2.09 Å).^[^ ^38^ ^]^

The thermal stability of both COFs was investigated by means of thermogravimetric analysis (TGA) (Figure S13, Supporting Information). The thermograms of COF PXX(PhNH_2_)_2_‐TFB and COF PXX(PhNH_2_)_2_‐Tp show a remarkable thermal stability, with values *T*_*d*10_ (thermal decomposition of 10% weight) of approximately 320 and 380 °C, respectively. Noteworthy, the thermal decomposition of both COFs is observed above 500 °C which is related to the cleavage of the imine bonds and the decomposition of COFs into oligomers and combustion products. The morphology and microstructure of the as‐prepared COFs materials were evaluated by means of scanning electron microscopy (SEM) (Figure S14 and S15, Supporting Information). It revealed that COF PXX(PhNH_2_)_2_‐Tp films are mainly made of amorphous domains (Figure S14, Supporting Information) whereas COF PXX(PhNH_2_)_2_‐TFB assemble forming spherical‐shaped particles with diameters ranging from 2 to 5 μm (Figure S15, Supporting Information).

Furthermore, the porosity and the Brunauer–Emmett–Teller (BET)‐specific surface areas of the COFs were determined through N_2_ adsorption–desorption isotherms recorded at 77 K (Figure S16, Supporting Information). Figure S16a (Supporting Information) reveals that COF PXX(PhNH_2_)_2_‐TFB possesses a lower N_2_ adsorption capacity, as evidenced by a BET surface area of 58 m^2^ g^−1^, in contrast to COF PXX(PhNH_2_)_2_‐Tp, which exhibits a higher value of 422 m^2^ g^−1^ (Figure S16b, Supporting Information). The total pore volumes of pores with a diameter less than 901 Å evaluated at *P*/*P*_0_ = 0.98 are 0.18 cm^3^ g^−1^ for COF PXX(PhNH_2_)_2_‐TFB and 0.682 cm^3^ g^−1^ for COF PXX(PhNH_2_)_2_‐Tp (Table S3, Supporting Information).

### 2.3 Electrochemical Characterization

#### 2.3.1 Three‐Electrode System

The electrochemical performance of COF PXX(PhNH_2_)_2_‐Tp and PXX(PhNH_2_)_2_‐TFB electrodes was first assessed in a three‐electrode system using 1 M zinc trifluoromethanesulfonate (Zn(CF_3_SO_3_)_2_) as aqueous electrolyte, Pt as counter electrode, Ag/AgCl as reference electrode, and the COF‐based electrodes as working electrodes. Figure S176a (Supporting Information) shows the cyclic voltammetry (CV) curves of both COFs at a scan rate of 10 mV s^−1^. The CV curve of PXX(PhNH_2_)_2_‐Tp displays a pair of redox peaks at 0.32/0.47 V, originating from the redox activity of the carbonyl groups of Tp, in agreement with other reported β‐ketoenamine‐linked COFs.^[^ ^39^ ^]^ At the same scan rate, the CV curve of PXX(PhNH_2_)_2_‐TFB is quasirectangular with no evident redox peaks, indicating that the charge storage mechanism of PXX(PhNH_2_)_2_‐TFB is dominantly capacitive. Figure S17b,c (Supporting Information) displays the CV curves of PXX(PhNH_2_)_2_‐Tp and PXX(PhNH_2_)_2_‐TFB, respectively, at different scan rates from 10 to 200 mV s^−1^. As the scanning potential increases, both the anodic and cathodic peak potentials of PXX(PhNH_2_)_2_‐Tp show minimal shifts toward the positive and negative directions, respectively. This suggests that there is no significant increase in ohmic resistance or polarization. On the other hand, the CV curves of PXX(PhNH_2_)_2_‐TFB remain symmetric even at the high scan rate of 200 mV s^−1^, indicating an excellent rate capability.

The specific capacitance of PXX(PhNH_2_)_2_‐Tp and PXX(PhNH_2_)_2_‐TFB amounts to 55 and 18 F g^−1^, respectively (see Experimental Section for details). By and large, the electrochemical performance of PXX(PhNH_2_)_2_‐Tp exceeds the one of PXX(PhNH_2_)_2_‐TFB in the three‐electrode system. The electrochemical characteristics of an electrode material are subjected to the influence of numerous physical properties, encompassing surface area, porosity, electronic conductivity, ionic conductivity, ion accessibility, crystal structure, redox‐active sites, and chemical stability. As can be seen in Table S4 (Supporting Information), although PXX(PhNH_2_)_2_‐Tp exhibits lower crystallinity than PXX(PhNH_2_)_2_‐TFB, it shows not only redox activity but also exhibits a higher surface area and total pore volume (422 m^2^ g^−1^ and 0.682 cm^3^ g^−1^, respectively) than PXX(PhNH_2_)_2_‐TFB (58 m^2^ g^−1^ and 0.18 cm^3^ g^−1^, respectively). These characteristics enhance accessibility to the redox‐active carbonyl groups of PXX(PhNH_2_)_2_‐Tp, subsequently boosting the electrode performance.

#### 2.3.2 Zn‐HSCs

To further investigate the electrochemical performance of the COF electrodes for practical applications as cathode materials in aqueous Zn‐HSCs, a single electrode with a mass loading of ≈1 mg cm^−2^ was assembled in a coin‐type cell with an Zn anode by using 4 M Zn(CF_3_SO_3_)_2_ as aqueous electrolyte. Figure S18a,b (Supporting Information) exhibits the CV curves of the first five cycles between 0 and 1.8 V at a scan rate of 10 mV s^−1^ of PXX(PhNH_2_)_2_‐Tp and PXX(PhNH_2_)_2_‐TFB, respectively. The CV curves of both COFs clearly show the characteristics of both the double‐layer capacitor and battery‐type electrode behavior.^[^ ^40^ ^]^ A pair of broad redox peaks at ≈1.50 V indicates the reversible adsorption/desorption processes of Zn^2+^ in the surface of the COF structures. Importantly, the peak positions remain almost unchanged during the successive cycling, implying that the electrochemical reactions occurring at the electrode's surface during the cycling process are highly reversible.

**Figure 2:**
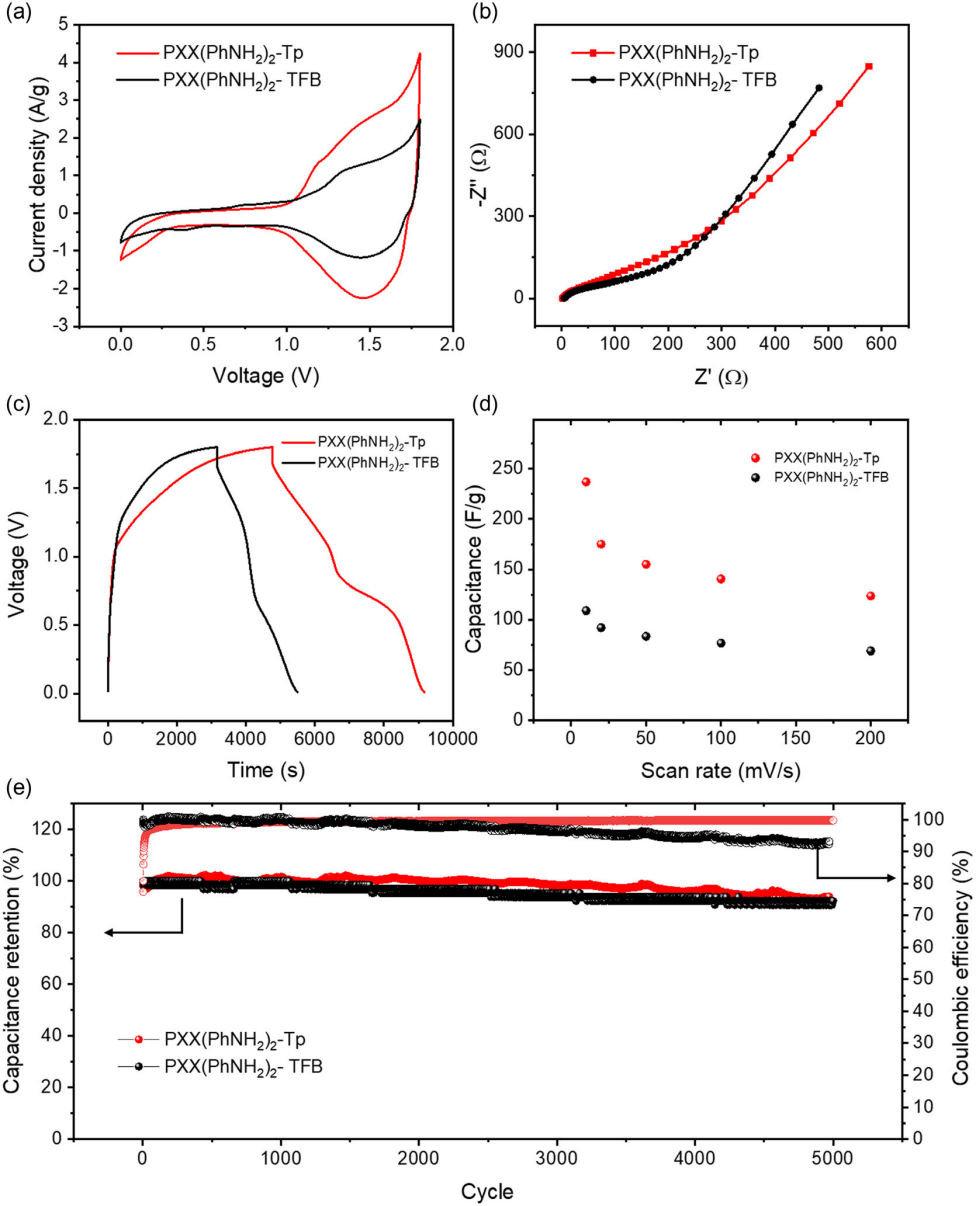
a) CV curves at 20 mV s^−1^ scan rate, b) Nyquist plots, and c) GCD profiles at 0.05 A g^−1^ of PXX(PhNH_2_)_2_‐Tp (red curves) and PXX(PhNH_2_)_2_‐TFB (black curves), d) specific capacitance of PXX(PhNH_2_)_2_‐Tp (red points) and PXX(PhNH_2_)_2_‐TFB (black points) at various scan rates, and e) long‐term cycling performance of PXX(PhNH_2_)_2_‐Tp (red points) and PXX(PhNH_2_)_2_‐TFB (black points) at 1 A g^−1^.

Electrochemical impedance spectroscopy (EIS) data were analyzed using Nyquist plots (**Figure** **2**b) and the experimental results were well fitted with the indicated circuit (Figure S19, Supporting Information). Both COF materials exhibit a low charge transfer resistance (*R*_ct_) (39.95 Ω for PXX(PhNH_2_)_2_‐Tp and 44.06 Ω for PXX(PhNH_2_)_2_‐TFB meaning that both materials allow for efficient and rapid transfer of charge (electrons or ions) between the electrode's surface and the electrolyte or the external circuit (Table S5, Supporting Information). The Zn^2+^ diffusion coefficients (D_Zn_^2+^) are obtained from the Warburg impedance in the low‐frequency region of the Nyquist plot in Figure S20 (Supporting Information). The estimated D_Zn_^2+^ amounts to 3.52 × 10^−13^ cm^2^ s^−1^ and 6.32 × 10^−13^ cm^2^ s^−1^ for PXX(PhNH_2_)_2_‐Tp and PXX(PhNH_2_)_2_‐TFB, respectively, meaning that both COFs exhibit a fast Zn^2+^ transport kinetics. The Zn^2+^ diffusion coefficient is higher in PXX(PhNH_2_)_2_‐TFB compared to PXX(PhNH_2_)_2_‐Tp, primarily owing to the superior crystalline structure of the former. However, it is imperative to emphasize that the crystallinity of a COF does not serve as a sole determinant of its electrochemical performance. It is essential to consider other relevant physical characteristics, as indicated in the three‐electrode system analysis.

The galvanostatic charge–discharge (GCD) profiles at 0.05 A g^−1^ of PXX(PhNH_2_)_2_‐Tp and PXX(PhNH_2_)_2_‐TFB (Figure 2c) in the explored potential window reveal a distorted triangular shape, in agreement with the hybrid nature of both COF electrodes (see Section 2.3.4 for details). The specific capacitances are calculated from the CV curves at different scan rates (Figure 2d), obtaining the highest value of 237 F g^−1^ for PXX(PhNH_2_)_2_‐Tp at the lowest scan rate, 10 mV s^−1^. This value is superior to COF PXX(PhNH_2_)_2_‐TFB, whose maximum specific capacitance amounts to 109 F g^−1^ at the same scan rate, and it is within the state of the art of the reported cathodes based on other organic and inorganic electrode materials in Zn‐HSCs (Table S6, Supporting Information). COF PXX(PhNH_2_)_2_‐Tp also displays a good rate capability, retaining a capacitance of 124 F g^−1^ at the high scan rate of 200 mV s^−1^. The Ragone plot of the prepared COF‐based electrodes (Figure S22, Supporting Information) displays a remarkable maximum energy density of 106.6 Wh kg^−1^ and power density of 3.0 kW kg^−1^ for PXX(PhNH_2_)_2_‐Tp electrodes. Table S6 (Supporting Information) shows that only a limited number of cathode materials exhibit a combination of high energy and power density, proving that COFs’ molecular design represents a viable strategy to obtain devices with high energy density (>95 Wh kg^−1^) and power density (>2 kW kg^−1^) from critical raw materials free sources. In particular, among the other reported COFs that serve as cathodes for Zn‐HSCs, there is only one example with superior energy and power densities than COF PXX(PhNH_2_)_2_‐Tp.^[^ ^41^ ^]^ However, the cyclability of this COF is inferior to the one of PXX(PhNH_2_)_2_‐Tp. Impressively, among the other reported electrodes for Zn‐HSCs only the system composed of activated carbon cathode and 2D Zn/Ni anode exhibits a better performance than COF PXX(PhNH_2_)_2_‐Tp.^[^ ^42^ ^]^ Overall, the performance of COF PXX(PhNH_2_)_2_‐Tp in terms of capacitance, energy, and power density and cyclability can be considered among the top of the state‐of‐the‐art cathodes for Zn‐HSCs.

#### 2.3.3 Postmortem Analysis

Long‐term stability of the prepared COF‐based electrodes was then investigated using galvanostatic charge–discharge cycles at the current density of 1 A g^−1^ (Figure 2e). PXX(PhNH_2_)_2_‐Tp and PXX(PhNH_2_)_2_‐TFB electrodes show an excellent long‐term stability with a capacitance retention exceeding 94% and 92%, respectively, over 5000 cycles, together with a coulombic efficiency of nearly 100%, indicating the better reversible supramolecular interaction with Zn^2+^ ions. To ascertain the stability of the electrodes, SEM, energy‐dispersive X‐ray spectroscopy (EDX), FTIR, and XPS analyses of PXX(PhNH_2_)_2_‐Tp cathode electrode after 5000 cycles were performed (Figure S23–S25, Supporting Information). Crucially, the morphology of PXX(PhNH_2_)_2_‐Tp cathodes does not show any obvious change after 5000 cycles, except for the surface incorporation of a zinc layer. This observation suggests that these cathodes exhibit remarkable stability throughout the charge‐discharge cycling. After 5000 charge/discharge cycles, the FTIR spectrum of COF PXX(PhNH_2_)_2_‐Tp (Figure S26, Supporting Information) exhibits the characteristic C═N stretching vibration and C—N enamine stretching at identical wavenumbers as the pristine COF (1612 and 1298 cm^−1^, respectively). To further confirm the chemical stability of COF PXX(PhNH_2_)_2_‐Tp, XPS analysis is then performed. Figure S27 (Supporting Information) depicts the high‐resolution spectra of C1s, N1s, and O1s for COF PXX(PhNH_2_)_2_‐Tp. Interestingly, the XPS spectra indicate no significant deviations from the pristine state in all cases, indicating the excellent chemical stability of COF PXX(PhNH_2_)_2_‐Tp during cycling.

#### 2.3.4 Charge Storage Mechanism

To gain insight into the charge storage mechanism of COF‐based materials, CV profiles of COF PXX(PhNH_2_)_2_‐Tp and PXX(PhNH_2_)_2_‐TFB at different scan rates were recorded (Figure S28a and S29a, Supporting Information). The CV curves only exhibit a small peak shift within the scan rate of 10–200 mV s^−1^, proving excellent electrochemical reversibility. The kinetics of the electrochemical processes occurring at both electrode materials are analyzed using the procedure proposed by Wu et al. (Equation (5)). The average values of *b* for COF PXX(PhNH_2_)_2_‐Tp and PXX(PhNH_2_)_2_‐TFB are 0.92 and 0.97, respectively, indicating that both COF‐based materials possess a hybrid nature with a charge storage mechanism predominantly ruled by a capacitive contribution, which is higher in the case of PXX(PhNH_2_)_2_‐TFB and partial diffusion‐controlled contribution (Figure S28b and S29b, Supporting Information). The higher diffusion‐controlled contribution for PXX(PhNH_2_)_2_‐Tp is substantiated by the existence of redox‐active carbonyl groups within the COF structure.

To further analyze the capacitive contribution and the diffusion‐controlled contribution at a specific scan rate, Equation (5) can be divided into two parts (Equation (6)). The capacitive and diffusion‐controlled capacity values at different rates are calculated and shown in Figure S30 (Supporting Information) for COF PXX(PhNH_2_)_2_‐Tp and Figure S31 (Supporting Information) for COF PXX(PhNH_2_)_2_‐TFB. At a scan rate of 10 mV s^−1^, around 76% of the total current in COF PXX(PhNH_2_)_2_‐Tp is governed by capacitive‐limited processes. At the same scan rate, around 80% of the total current in COF PXX(PhNH_2_)_2_‐TFB is governed by capacitive‐limited processes. As the scan rate increases, the contribution ratio of the capacitive process gradually rises for both COF‐based materials (Figure S30a and S31a, Supporting Information). Notably, at the highest scan rate (200 mV s^−1^), PXX(PhNH_2_)_2_‐TFB exhibits the highest capacitive contribution of 95% to the charge storage mechanism. Hence, the presence of both capacitive and diffusion‐controlled charge storage mechanisms confirms the hybrid nature of COF PXX(PhNH_2_)_2_‐Tp and PXX(PhNH_2_)_2_‐TFB materials, offering significant benefits for energy storage applications.

A potential hybrid charge storage mechanism is proposed as follows: Zn^2+^ ions may coordinate with the oxygen heteroatoms of the PXX(PhNH_2_)_2_ units in both COFs via a capacitive charge storage mechanism. Moreover, in COF PXX(PhNH_2_)_2_‐Tp, Zn^2+^ ions could also interact with the redox‐active carbonyl groups in the β‐ketoenamine form. To access both the oxygen heteroatoms and carbonyl groups, Zn^2+^ ions must diffuse through the COF structure, employing a diffusion charge storage mechanism. Concurrently, Zn^2+^ ions may also be stored in accessible interstitial sites at the COF interface or pores, or within the carbonyl units of the organic building unit in COF PXX(PhNH_2_)_2_‐Tp, via a capacitive charge storage mechanism. As indicated by the CV analysis, the dominant charge storage mechanism in both COFs is capacitive, indicating that ions are stored at the interface between the electrode and the electrolyte in a double‐layer capacitance fashion or that the charge storage is not limited by ion diffusion. The significant porosity of COF PXX(PhNH_2_)_2_‐Tp poses no hindrance to the diffusion of Zn^2+^ ions through the COF structure, enabling their intercalation and effective interaction with the carbonyl groups.

To cast further light onto the charge storage mechanism of Zn^2+^ ions by the COF PXX(PhNH_2_)_2_‐Tp and COF PXX(PhNH_2_)_2_‐TFB cathodes, XPS analyses were performed in the pristine, fully charged (1.8 V) and fully discharged (0 V) states (Figure S32–S39, Supporting Information). The high‐resolution Zn 2p spectra (Figure S32b and S33b, Supporting Information) reveal the typical peaks at 1021 and 1044 eV of Zn^2+^, which exhibit a higher intensity in the discharged state for both cathode materials as a consequence of their coordination with the COF electrodes. The high‐resolution C1s and O1s spectra of COF PXX(PhNH_2_)_2_‐Tp (Figure S34 and S38, Supporting Information) display that the peak intensities of C═O and C—O change cyclically during the electrochemical reactions, which further confirms the redox‐active nature of the carbonyl groups. Conversely, there are no significant distinctions observed in the high‐resolution C1s and O1s spectra of COF PXX(PhNH_2_)_2_‐TFB primarily because of its inherent lack of redox activity (Figure S35 and S39, Supporting Information).

## 3 Conclusion

In summary, we have developed and characterized two unprecedented COFs, namely, PXX(PhNH_2_)_2_‐Tp and PXX(PhNH_2_)_2_‐TFB, with the intention of utilizing them as cathode materials in Zn‐HSCs. These COFs feature different structural characteristics and bonding types. PXX(PhNH_2_)_2_‐Tp exhibited a larger surface area (422 m^2^ g^−1^), higher pore volume (0.682 cm^3^ g^−1^), and contained both imine and β‐ketoenamine forms. In contrast, PXX(PhNH_2_)_2_‐TFB exhibited solely imine bonds and a lower surface area (58 m^2^ g^−1^) and pore volume (0.18 cm^3^ g^−1^). As cathode materials in Zn‐HSCs, PXX(PhNH_2_)_2_‐Tp outperforms PXX(PhNH_2_)_2_‐TFB and competes with other reported cathode materials, achieving a specific capacitance of 237 F g^−1^ at a low scan rate of 10 mV s^−1^, high energy density (107 Wh kg^−1^), and power density (3.0 kW kg^−1^). Impressively, both COFs displayed outstanding chemical stability, with over 92% capacitance retention after 5000 charge/discharge cycles. The analysis of the charge storage mechanism indicates that both COFs possess a hybrid nature, involving capacitive and diffusion‐controlled charge storage. PXX(PhNH_2_)_2_‐Tp shows a higher diffusion‐controlled contribution, attributed to its redox‐active carbonyl groups. These findings highlight the potential of PXX‐based COFs as high‐performance cathode materials for energy storage applications, particularly in Zn‐HSCs. Besides, the current synthetic protocols show a promise for industrial scalability, enhancing COFs’ commercial viability. More generally, this study showcases the role of COF design in tailoring electrochemical performance, emphasizing the importance of surface area, pore characteristics, and redox‐active groups in enhancing Zn‐HSC cathode materials.

## 4 Experimental Section

4.1

4.1.1

##### Material Characterization

The composition, structure, and texture properties of materials were investigated by PXRD patterns (Bruker D8 X‐ray diffractometer). TGA decomposition curves were recorded in the range 25–300 °C operating under air or nitrogen atmosphere, with a thermal step of 10 °C min^−1^ on a Mettler Toledo TGA/SDTA851e system. XPS (Thermo Scientific K‐Alpha X‐ray photoelectron spectrometer) equipped with an aluminum X‐ray source (energy 1.4866 keV) at a vacuum level of 10^−8^–10^−9^ mbar in the main chamber. The spot size of the X‐ray beam was fixed at 400 μm. The specific surface area was measured using a Micromeritics ASAP 2050 surface area and porosity analyzer. Before the nitrogen sorption measurements, the samples were outgassed for 12 h at 95 °C. Adsorption isotherms were calculated for nitrogen adsorption at 77 K and pressure up to 1 bar. SEM images and EDX were recorded with a FEI Quanta FEG 250 instrument S3 (FEI corporate, Hillsboro, Oregon, USA). NMR spectra were recorded on a Bruker Fourier 500 MHz spectrometer equipped with a dual (^13^C, ^1^H) probe. Chemical shifts were reported in ppm relative to tetramethylsilane using the solvent residual signal as an internal reference DMSO‐d6: *δ*_H_ = 2.50 ppm, *δ*_C_ = 39.52 ppm. Coupling constants (*J*) were given in Hz. Resonance multiplicity was described as *s* (singlet), *d* (doublet), *t* (triplet), *q* (quartet), and broad (broad signal). Carbon spectra were acquired with a complete decoupling for the proton. ssNMR ^13^C NMR spectra were recorded on a Bruker Avance III HD spectrometer coupled with an 11.7 T wide bore superconducting magnet operating at 125.76 MHz ^13^C Larmor frequency. All spectra were recorded at 298 K stabilized temperature using the magic‐angle spinning technique for high‐resolution NMR spectroscopy in the solid state using 4 mm rotors. The spinning frequency was 10 kHz. IR spectra were recorded on a Shimadzu IR Affinity 1S FTIR spectrometer in ATR mode with a diamond monocrystal. Selected absorption bands are reported by wavenumber (cm^−1^).

##### Computational Methods

Geometrical optimization of isolated structures of Tp, TFB, and PXX(PhNH_2_)_2_ was performed using DFT at the M062X/6‐311++G** level, followed by analysis of their vibrational frequencies to characterize the structures obtained as energy minima. These computations were performed using Gaussian16. The description for the vibrational modes and its assignment to the theoretical IR spectra was performed by visual inspection of the atomic displacements for each vibrational mode.

##### Electrochemical Measurements

The electrochemical performance of PXX(PhNH_2_)_2_‐Tp and PXX(PhNH_2_)_2_‐TFB electrodes was studied using CV, and EIS on Autolab PGSTAT128N Potentiostat/Galvanostat instruments with a Metrohm Autolab DuoCoin Cell Holder (Metrohm AG) at room temperature. CV was performed at scan rates of 10–200 mV s^−1^ in the voltage range between 0 and 1.8 V or 0 and 1.2 V. EIS measurement was recorded with a frequency range of 0.01 Hz to 1 MHz. The GCD tests were carried out on the Neware Battery Tester (BTS‐4008T‐5V/10 mA, Neware Technology Company, Guangdong, China). GCD curves were tested at current densities ranging from 0.05 to 1 A g^−1^.

##### Electrodes Preparation and Electrochemical Measurements: Fabrication of Working Electrodes

The working electrodes were prepared by mixing of COFs (80% wt, 16 mg), carbon black (10% wt, 2 mg), and PVDF (10% wt, 2 mg) in agate mortar with several drops of NMP to obtain a homogenous paste. The paste was then coated into a carbon paper electrode with a diameter of 0.8 cm and further dried under vacuum at 80 °C. The mass loading of the COFs was ≈1 mg cm^−2^ in each electrode. For the three‐electrode system, the paste was coated onto a Ni foam electrode.

##### Electrodes Preparation and Electrochemical Measurements: Fabrication of Three‐Electrode Configuration

1 M Zn(CF_3_SO_3_)_2_ aqueous solution served as electrolyte, Pt as counter electrode, Ag/AgCl as reference electrode, and COF‐based electrodes as working electrodes.

##### Electrodes Preparation and Electrochemical Measurements: Fabrication of Two‐Electrode Configuration

Two electrodes were assembled in CR2032 stainless steel coin‐type cells with a porous cellulose membrane as separator 4 M Zn(CF_3_SO_3_)_2_ as electrolyte.

##### Electrochemical Calculations: Calculation of the Specific Capacitances

The specific capacitance was calculated using GCD with following equation:^[^ ^43^, ^44^, ^45^ ^]^

(1) $$C_{s}=\frac{\;1}{\Delta V\;\cdot \;m\cdot s}\;\cdot \;\int I\left(V\right)dV$$

where Δ*V* (V) is the voltage window and *m* (g) is the mass of COFs in a single electrode, *s* is the scan rate (V s^−1^), and ∫I(V)dV represents the integral area of the CV curve.^[^ ^43^, ^46^ ^]^

##### Electrochemical Calculations: Power and Energy Density Calculations

The energy density of the device was obtained from the formula:

(2) $$E=\frac{I}{2\cdot m}\cdot \int_{0}^{t}V\cdot \Delta t$$

The power density of the device was calculated from the formula:

(3) $$P=\frac{E}{\Delta t}\cdot \;3600$$

where *E* is specific energy (Wh kg^−1^), *I* is the applied current (A), $\int_{0}^{t}V\cdot \Delta t\;$ is the integral area of the GCD curve, *P* is the power density (W kg^−1^), and Δ*t* is the discharge time (s).

##### Electrochemical Calculations: Calculation of the Zn^2+^ Diffusion Coefficient (D_Zn_^2+^)

(4) $$D_{Zn^{2+}}^{EIS}=\frac{R^{2}\cdot T^{2}}{2\cdot A^{2}\cdot n^{4}\cdot F^{4}\cdot c^{2}\cdot \delta^{2}}$$

where *R* is the gas constant (8.314 J (mol K)^−1^), *T* is the absolute temperate (298 K), *A* is the surface area of the electrode (0.5 cm^2^), *n* is the electron‐transfer number (1), *F* is the Faraday constant (96 500 C mol^−1^), *c* is the concentration of Zn^2+^ in the electrode (0.004 mol cm^−3^), and *δ* is the Warburg coefficient derived from the linear relation between *ω* ^−1/2^ and *Z′* (158.55 and 118.3 Ω s^−1/2^ for COF PXX(PhNH_2_)_2_‐Tp and COF PXX(PhNH_2_)_2_‐TFB, respectively.

Calculations for the kinetics of the electrochemical processes are occurring at both electrode materials:

(5) $$i\;=\;a\nu^{b}$$

where *i* is the peak current and *ν* is the scan rate.

(6) $$i\;=\;k_{1}\nu \;+\;k_{2}\nu^{1/2}$$

where *k*_1_ *ν* and *k*_2_ *ν* ^1/2^ represent the capacitive and diffusion limited effects, respectively.

## Conflict of Interest

The authors declare no conflict of interest.

## Supporting information

Supplementary Material

## Data Availability

The data that support the findings of this study are available from the corresponding author upon reasonable request.
