# Supplementary material for: Peri‐Xanthenoxanthene‐Based Covalent Organic Frameworks for High‐Performance Aqueous Zn‐Ion Hybrid Supercapacitors
Source: Small Sci. 2024 Apr 26;4(7):2400031. doi: 10.1002/smsc.202400031 (PMC11935269; doi:10.1002/smsc.202400031)
Supplement: Supplementary file 1 — Supplementary Material [file SMSC-4-2400031-s001.pdf]

Supporting Information

**Peri-Xanthenoxanthene-based covalent organic frameworks for high-performance aqueous Zn-ion hybrid supercapacitors**

*Cataldo Valentini, Verónica Montes-García, Luca Cusin, Dawid Pakulski, Mateusz Wlazło, Paolo Samorì,* and Artur Ciesielski**

Dr. C. Valentini, Dr. V. Montes-García, Dr. L. Cusin, Prof. Dr. P. Samorì, Dr. A. Ciesielski
Université de Strasbourg and CNRS, ISIS, 8 allée Gaspard Monge, 67000 Strasbourg, France. E-mail: ciesielski@unistra.fr, samori@unistra.fr

Dr. C. Valentini, Dr. D. Pakulski, visiting Prof. Dr. A. Ciesielski
Centre for Advanced Technologies, Adam Mickiewicz University, Uniwersytetu Poznańskiego 10, 61-614 Poznań, Poland

Dr. M. Wlazło
Chemical and Biological Systems Simulation Lab, Centre of New Technologies, University of Warsaw, 02-097 Warsaw, Poland

## Table of context

**1. Synthesis of the monomer and COFs ........ 2**
**1.1 Synthesis of PXX. ........ 2**
**1.2 Synthesis of PXX-$Br_2$. ........ 2**
**1.3 Synthesis of PXX($PhNHBoc)_2$ ........ 2**
**1.4 Synthesis of COF PXX($PhNH_2)_2$-TFB ........ 4**
**1.5 Synthesis of COF PXX($PhNH_2)_2$-Tp ........ 4**
**2. DFT calculations ........ 6**
**3. COFs physical characterization ........ 7**
**3.3 X-ray diffraction (XRD) simulations ........ 10**
**3.6 Nitrogen sorption measurements ........ 15**
**4 Electrochemical characterization ........ 16**
**4.1 Three-electrode system ........ 16**
**4.2 Cyclic voltammetry ........ 18**
**4.3 Electrochemical Impedance Spectroscopy ........ 19**
**4.4 Galvanostatic charge discharge ........ 20**
**4.5 Post-mortem analysis ........ 24**
**4.6 Charge storage mechanism ........ 29**

5 References.....41

# 1. Synthesis of the monomer and COFs

## Synthetic procedures

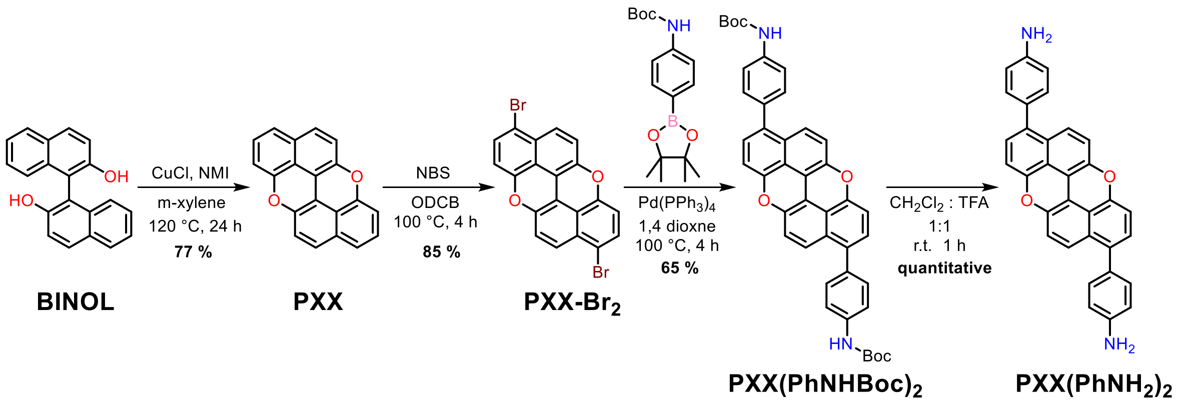


**Scheme S1**. Synthetic path for the synthesis of **PXX(PhNH$_2$)$_2$**

### 1.1 **Synthesis of PXX.**

This compound was prepared according to a modified literature procedure.[1]

### 1.2 **Synthesis of PXX-Br$_2$.**

This compound was prepared according to a modified literature procedure.[2]

### 1.3 **Synthesis of PXX(PhNHBoc)$_2$**

A solution of PXX-Br$_2$ (400 mg, 0.1 mmol, 1.0 eq.), 4-(N-Boc-amino)phenylboronic acid pinacol ester acid pinacol ester (696 mg, 2.18 mmol, 2.4 eq.), $K_2CO_3$ (691 mg, 5 mmol, 5.5 eq.) and $Pd(PPh_3)_4$ (53 mg, 0.045 mmol, 5 mol%) in 30 mL 1,4-dioxane and 5 mL $H_2O$ was degassed bubbling $N_2$ for 1 hour heated to reflux (110 °C) for 3 d. After cooling to room temperature, $H_2O$ was added. The aqueous phase was extracted with $CH_2Cl_2$ (25 mL x 3) and the combined organic phase dried on $Na_2SO_4$ and concentrated in vacuo. The crude residue was further purified in a silica gel column chromatography ($CH_2Cl_2$) yielding **PXX(PhNHBoc)$_2$** as a yellow solid (445.00 mg, 74%). IR (neat): cm$^{-1}$ 579.8, 765.7, 818.4, 938.1, 1017.9, 1055.4, 1152.7, 1229.4, 1239.7, 1310.8, 1369.0, 1396.1, 1467.2, 1494.3, 1525.4, 1579.7, 1614.7, 1696.2, 2926.7, 2968.2, 2988.9, 3051.2, 3388.7. $^1$H-NMR (500 MHz, DMSO) 9.50 (s, 2H), 7.59 (d, J = 8.2 Hz, 4H), 7.42 (d, J = 9.4 Hz, 2H), 7.33 (d, J = 8.3 Hz, 4H), 7.10 (d, J = 9.1 Hz, 4H), 6.85 (d, J = 7.9 Hz, 2H), 1.51 (s, 18H).$^{13}$C-NMR (126 MHz, DMSO) δ

153.3, 151.3, 144.2, 142.7, 139.4, 133.1, 132.8, 129.8, 129.2, 121.4, 118.7, 118.1, 111.2, 109.6, 28.6. HRMS (ES+): m/z $[M+H]^+$ calcd for ($C_{42}H_{37}N_2O_6$): 664.2573; found: 665.2612

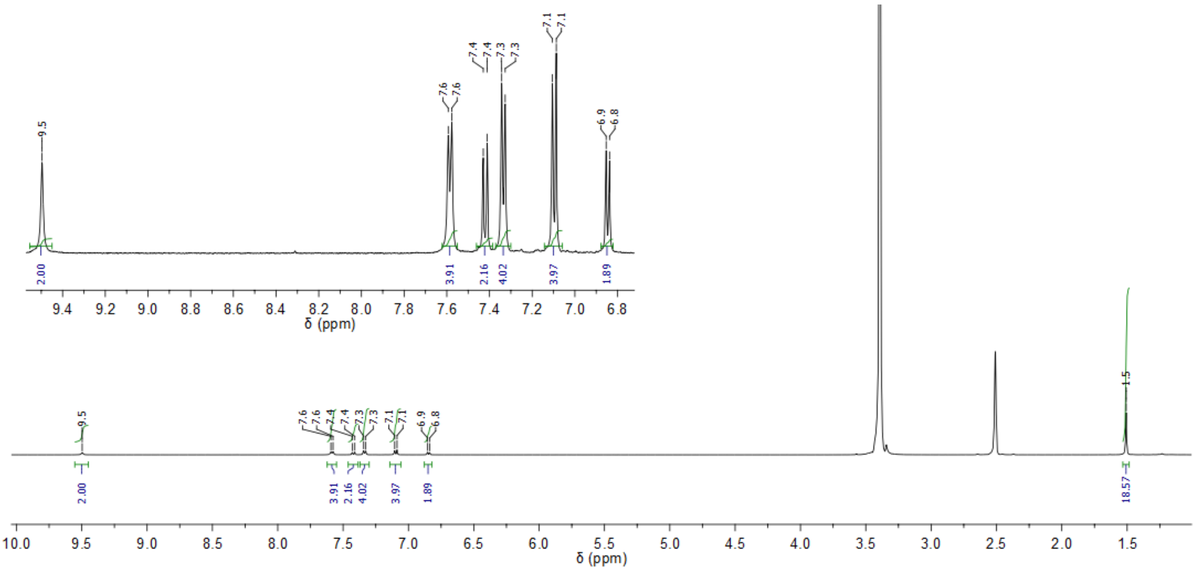


**Figure S1.** $^{1}$H-NMR, 500 MHz, DMSO-$d_6$

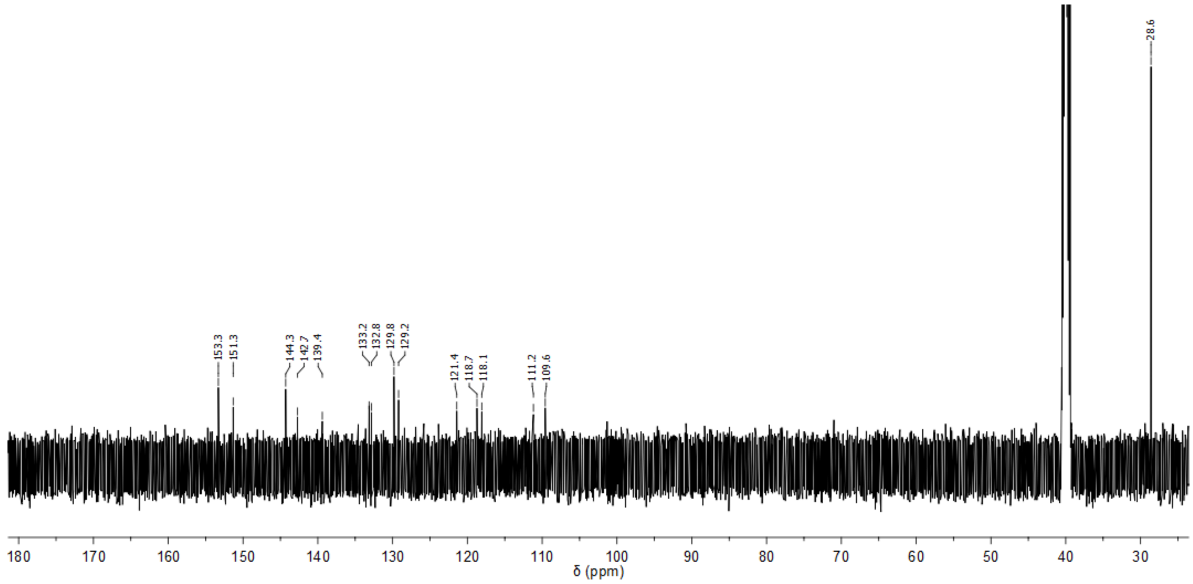


**Figure S2.** $^{13}$C-NMR, 125 MHz, DMSO-$d_6$

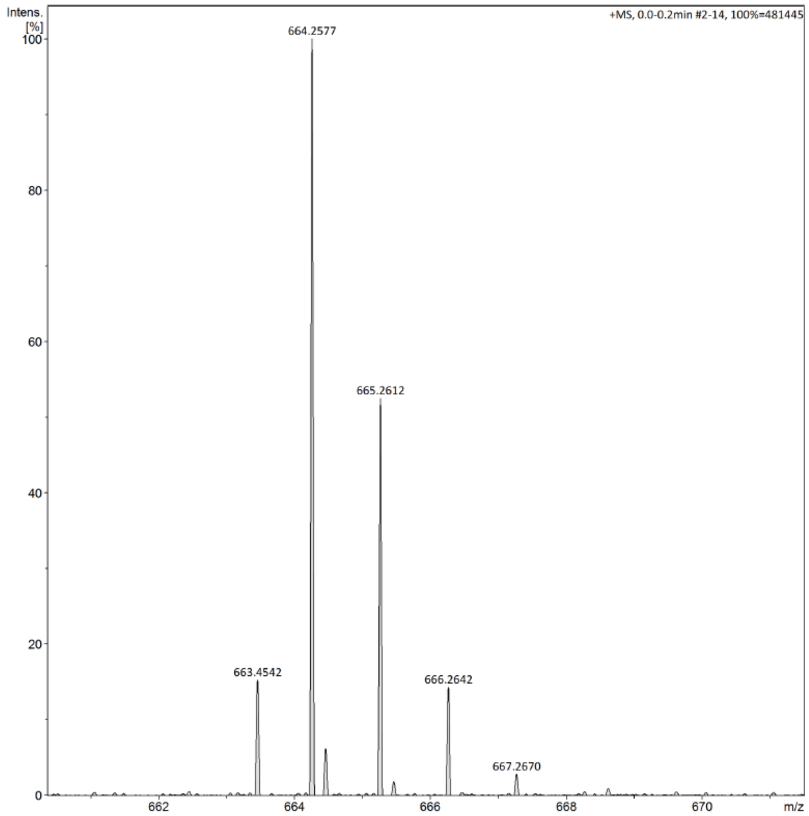


**Figure S3.** High resolution ESI-MS positive mode.

## 1.4 **Synthesis of COF PXX($PhNH_2$)$_2$-TFB**

To a solution of PXX(PhNHBoc)$_2$ (0.135 mol, 90 mg) in 8 mL of $CH_2Cl_2$ was added 2 mL of trifluoroacetic acid (TFA) under nitrogen and the solution stirred for 2 hours at room temperature. The solvent and TFA were evaporated through gently $N_2$ bubbling. The reaction crude was washed twice with 3 mL of toluene, evaporating the solvent bubbling $N_2$ every cycle, yielding PXX($PhNH_2$)$_2$. The product was dissolved in 9 mL of 1,4-dioxane and 9 mL of mesitylene and the tris-aldehyde (15 mg, 0.09 mmol) added. The reaction mixture was sonicated for 10 minutes and 2.4 mL of acetic acid 6 M added. The solution was degassed through freeze-pump-thaw (3 x 15 minutes) and heated at 120 °C for 5 days. The reaction crude was collected by filtration, washed with THF (10 mL) and purified via Soxhlet extraction with THF for 24 hours giving COF PXX($PhNH_2$)$_2$-TFB as a yellow powder in 47% yield (34 mg).

## 1.5 **Synthesis of COF PXX($PhNH_2$)$_2$-Tp**

To a solution of PXX(PhNHBoc)$_2$ (0.135 mol, 90 mg) in 8 mL of $CH_2Cl_2$ was added 2 mL of TFA under nitrogen and the solution stirred for 2 hours at room temperature. The solvent and

TFA were evaporated through gently $N_2$ bubbling. The reaction crude was washed twice with 3 mL of toluene, evaporating the solvent bubbling $N_2$ every cycle, yielding PXX(PhNH$_2$)$_2$. The product was dissolved in 9 mL of 1,2-dichlorobenzene and 9 mL of n-butanol and the tris-aldehyde (19 mg, 0.09 mmol) added. The reaction mixture was sonicated for 10 minutes and 2.4 mL of acetic acid 6 M added. The solution was degassed through freeze-pump-thaw (3 x 15 minutes) and heated at 120 °C for 5 days. The reaction crude was collected by filtration, washed with THF (10 mL) and purified via Soxhlet extraction with THF for 24 hours giving COF PXX(PhNH$_2$)$_2$-Tp as a red powder in 51% yield (41 mg).

## 2. DFT calculations

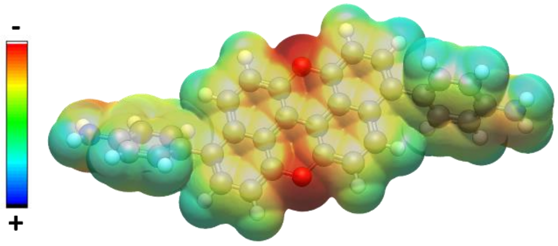


**Figure S4.** Electrostatic potential map of $PXX(PhNH_2)_2$.

## 3. COFs physical characterization

### 3.1 Fourier-Transform Infrared Spectroscopy (FTIR)

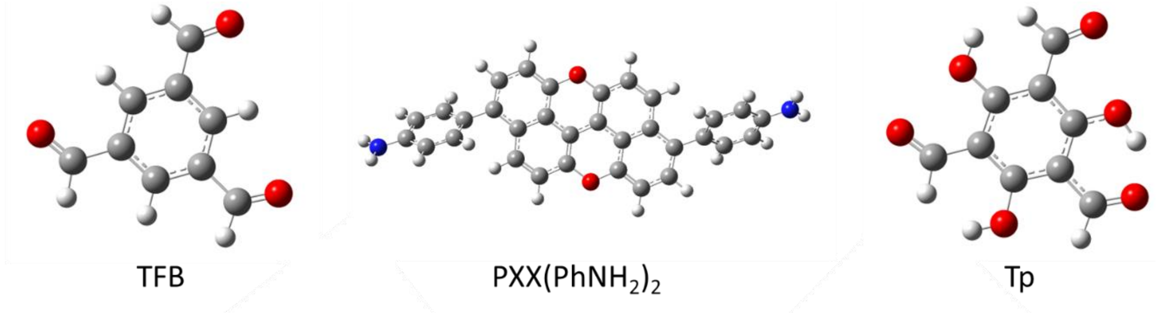


**Figure S5.** Chemical structures used for the Gaussian modelling.

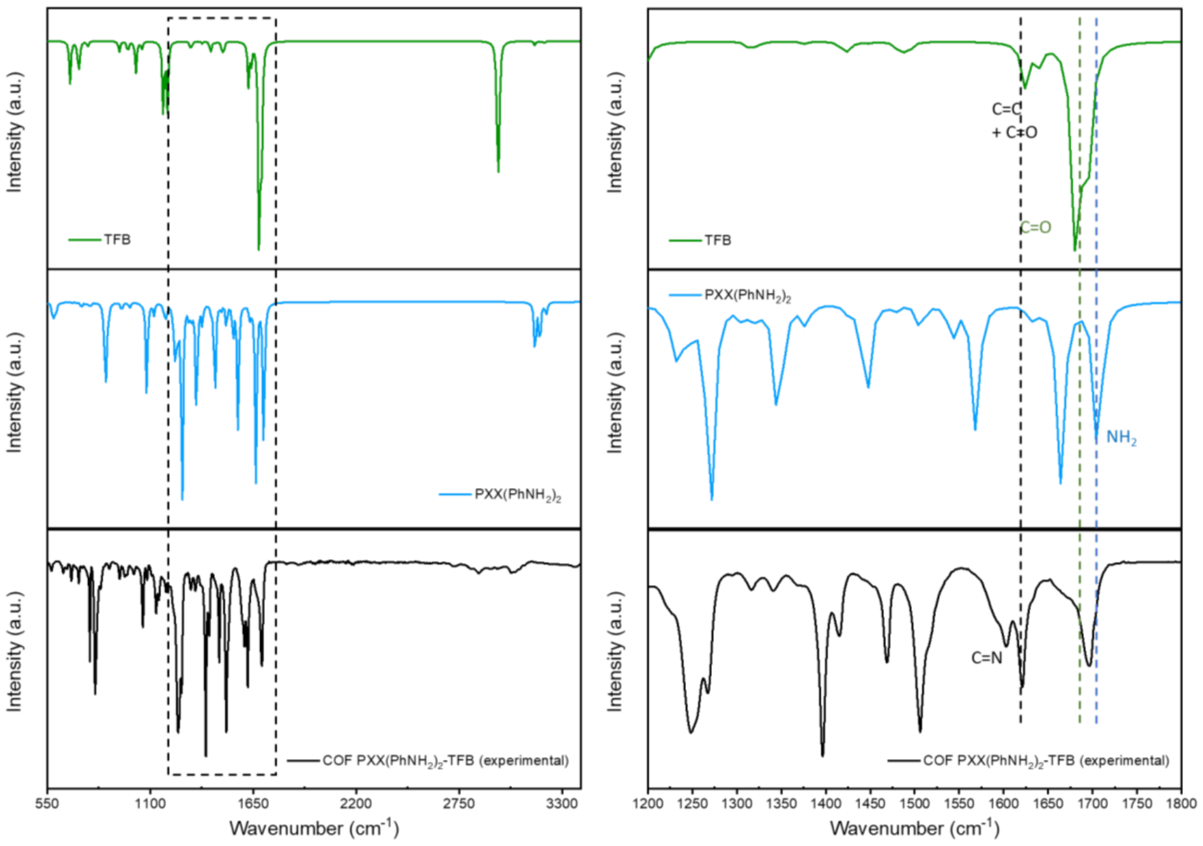


**Figure S6** Simulated FTIR spectra of TFB (green), $PXX(PhNH_2)_2$ (blue) and experimental FTIR spectrum of COF $PXX(PhNH_2)_2$-TFB (black).

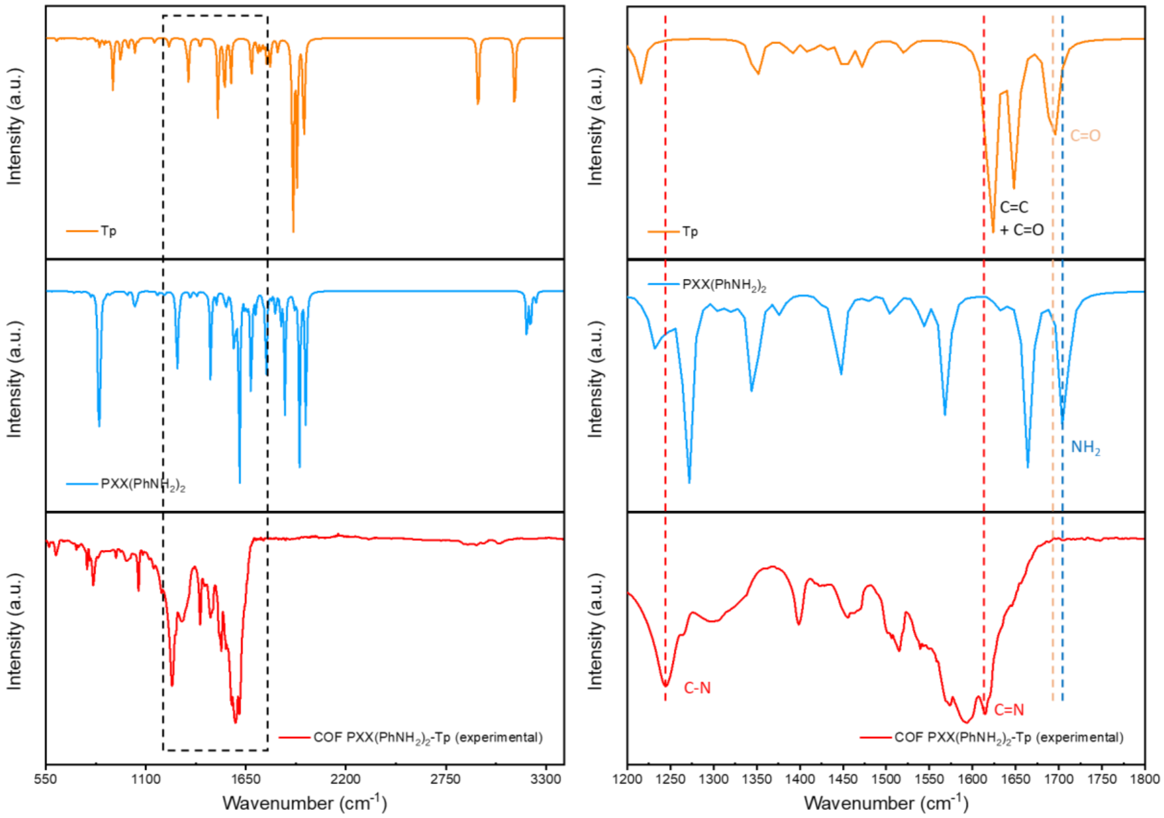


**Figure S7.** Simulated FTIR spectra of Tp (orange), $PXX(PhNH_2)_2$ (blue), repetitive unit of COF $PXX(PhNH_2)_2$-Tp (purple) and experimental FTIR spectrum of COF $PXX(PhNH_2)_2$-Tp (red).

### 3.2 X-ray photoelectron spectroscopy (XPS)

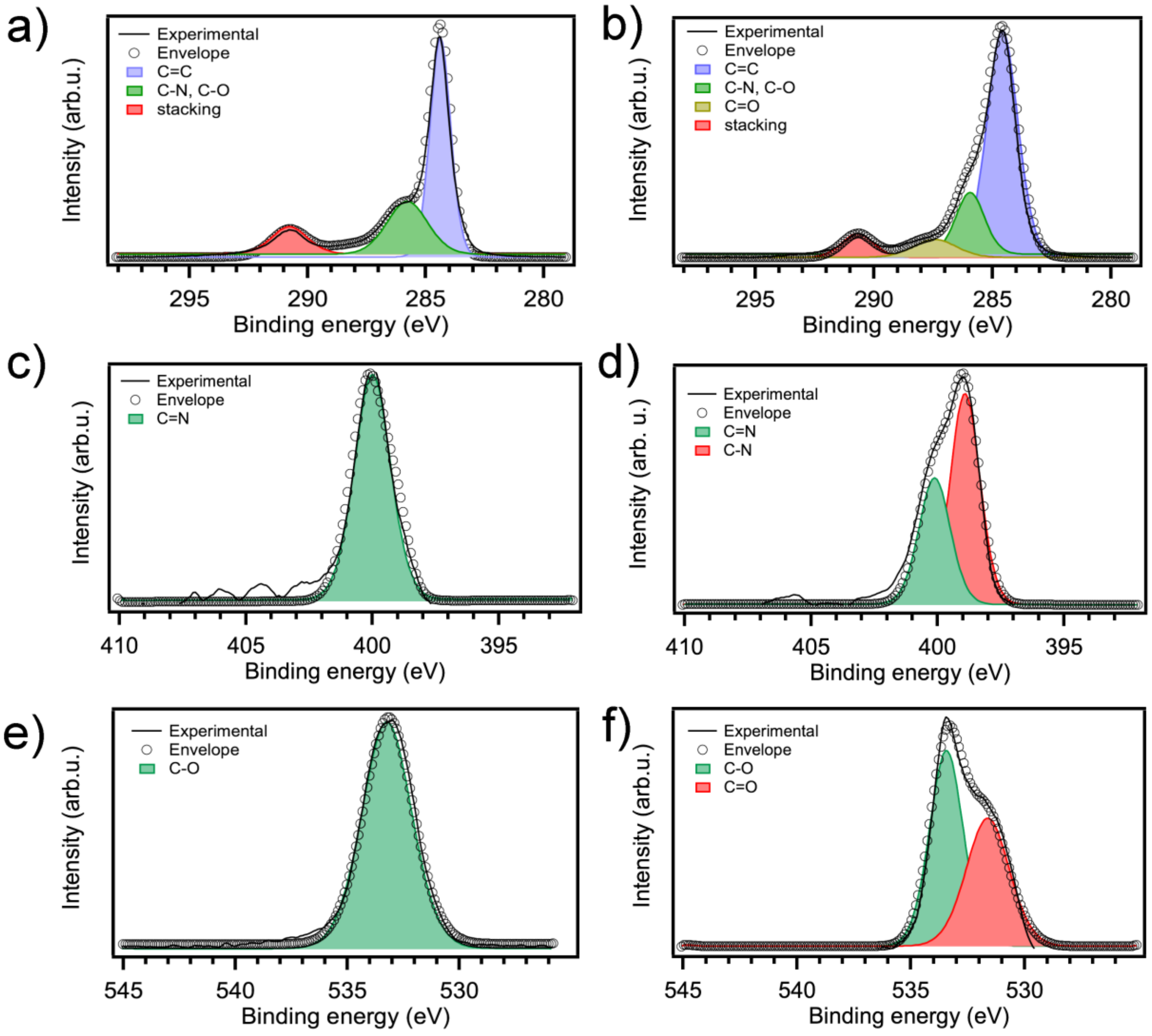


**Figure S8.** XPS of COF PXX($PhNH_2$)$_2$-TFB (left column) and COF PXX($PhNH_2$)$_2$-Tp (right column), a-b) C1s, c-d) N1s and e-f) O1s.

### 3.3 X-ray diffraction (XRD) simulations

Initially, a force field calculation was carried out in order to reach a state closer to the global minimum. After the initial relaxation, a planewave DFT calculation was performed assuming a 2D monolayer crystal. The level of theory used was DFT-PBE/D3(BJ). Due to periodic boundary conditions, a layer of vacuum was introduced above the monolayer to eliminate interactions between neighboring unit cells. The monolayer system was relaxed until forces acting on each atom vanished below 0.05 eV/Å. Then, the relaxed monolayer was used to construct the stacked structures (formula $C_{228}N_{12}O_{12}H_{120}$, twice the monolayer). Eclipsed (AA) and staggered (AB) stacking were considered. The AA and AB structures were relaxed again and converged to the same accuracy as the monolayer structure. The whole system, including unit cell and atoms, was allowed to relax. The interlayer distances and stacking energies for AA and AB configurations of both COFs are displayed in Table S2-3.

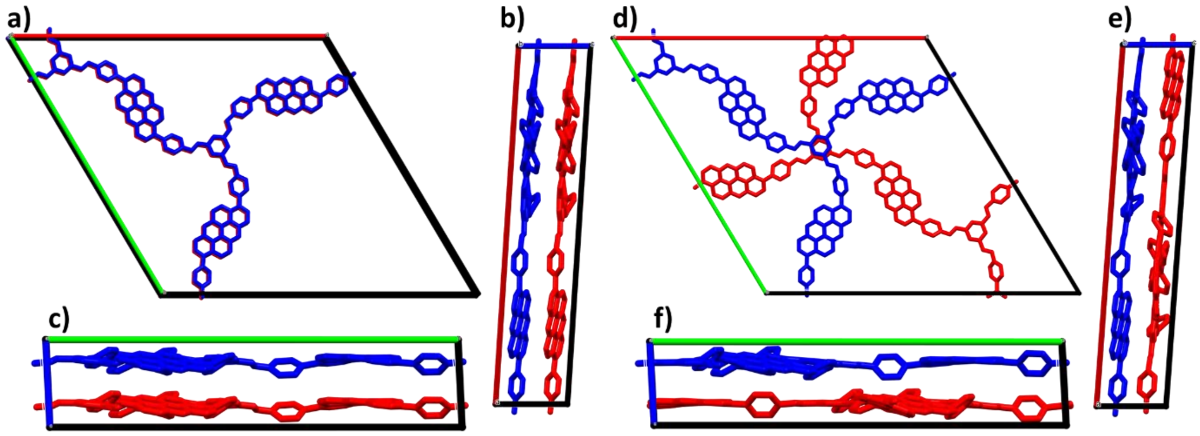


**Figure S9.** Simulated XRD structure of COF PXX($PhNH_2$)$_2$-TFB. a) view of AA symmetry along c; b) view of AA symmetry along b; c) view of AA symmetry along a; d) view of AB symmetry along c; e) view of AB symmetry along b; f) view of AB symmetry along a.

**Table S1.** Unit cell parameters of calculated COF PXX($PhNH_2$)$_2$-TFB.

| Monolayer | Eclipsed (AA) | Staggered (AB) |
|---|---|---|
| H60 C114 N6 O6 | H120 C228 N12 O12 | H120 C228 N12 O12 |
| Space Group: P 1 (#1-1) | Space Group: P 1 (#1-1) | Space Group: P 1 (#1-1) |
| a = 46.09426 Å α = 90.1220° | a = 44.14382 Å α = 89.0573° | a = 44.79785 Å α = 89.0635° |
| b = 43.67754 Å β = 90.3647° | b = 41.72429 Å β = 92.6874° | b = 42.46034 Å β = 93.1023° |
| c = 18.60635 Å γ = 59.7242° | c = 7.59991 Å γ = 59.2407° | c = 7.37677 Å γ = 59.0674° |
| V = 32350 Å$^3$ | V = 12002.3 Å$^3$ | V = 12002.3 Å$^3$ |
| | -168.36 kcal/mol | -49.13 kcal/mol |
| | d = 0.38 nm | d = 0.369 nm |

d : interplanar distance

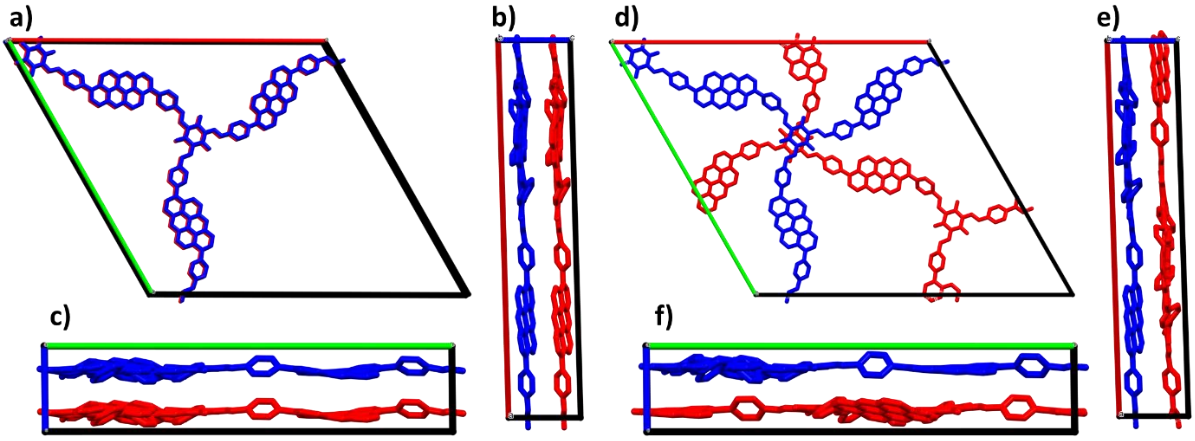


**Figure S10.** Simulated XRD structure of COF PXX$(PhNH_2)_2$-Tp. a) view of AA symmetry along c; b) view of AA symmetry along b; c) view of AA symmetry along a; d) view of AB symmetry along c; e) view of AB symmetry along b; f) view of AB symmetry along a.

**Table S2.** Unit cell parameters of calculated COF PXX$(PhNH_2)_2$-Tp.

| **Monolayer** | **Eclipsed (AA)** | **Staggered (AB)** |
|---|---|---|
| H60 C114 N6 O12 | H120 C228 N12 O24 | H120 C228 N12 O24 |
| Space Group: P 1 (#1-1) | Space Group: P 1 (#1-1) | Space Group: P 1 (#1-1) |
| a = 46.12901 Å α = 89.9255° | a = 45.63175 Å α = 88.3553° | a = 46.20701 Å α = 88.2678° |
| b = 43.61830 Å β = 90.1667° | b = 41.73502 Å β = 87.7957° | b = 42.38850 Å β = 87.5469° |
| c = 17.64668 Å γ = 60.1896° | c = 7.57486 Å γ = 60.2746° | c = 7.37365 Å γ = 60.1770° |
| V = 30101.3 Å$^3$ | V = 12517.6 Å$^3$ | V = 12517.6 Å$^3$ |
| | -191.59 kcal/mol | -45.45 kcal/mol |
| | d = 0.379 nm | d = 0.369 nm |

d : interplanar distance

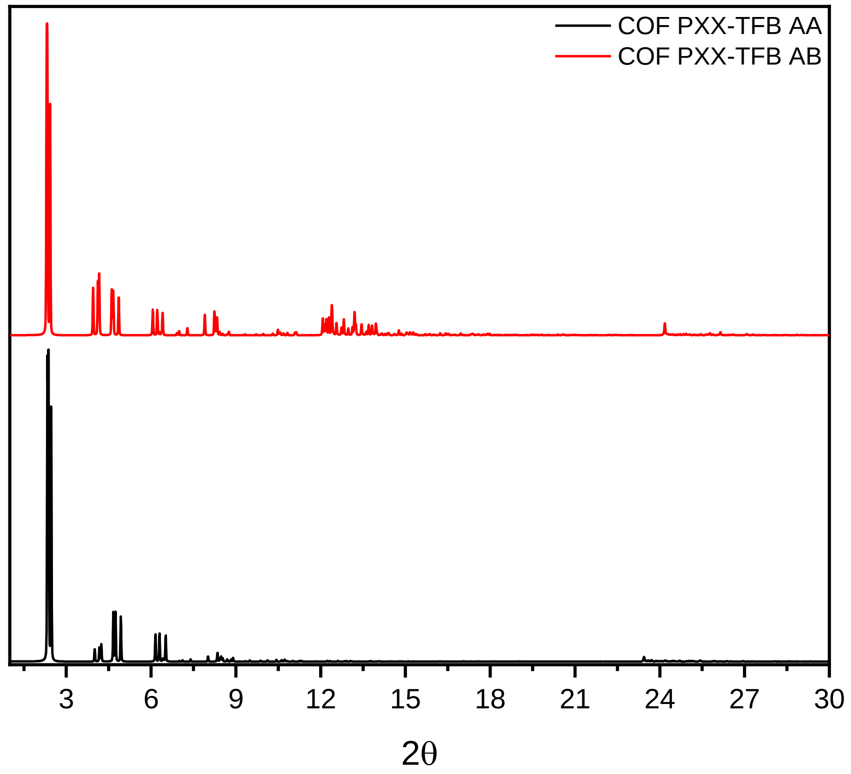


**Figure S11.** Simulated XRD pattern of COF PXX($PhNH_2$)$_2$-TFB.

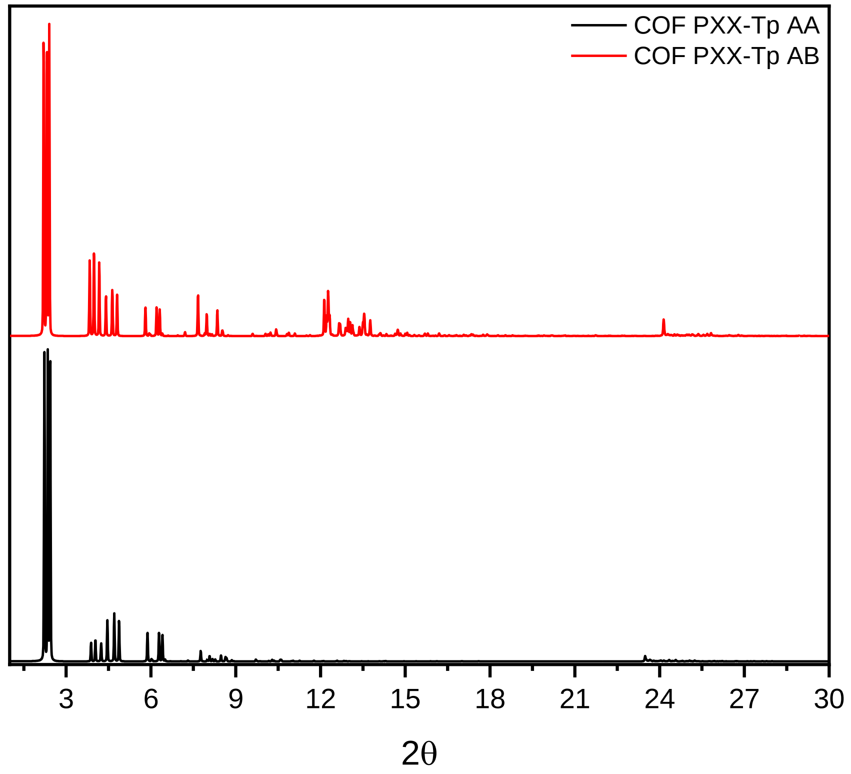


**Figure S42.** Simulated XRD pattern of COF PXX($PhNH_2$)$_2$-Tp.

### 3.4 Thermogravimetric analysis (TGA)

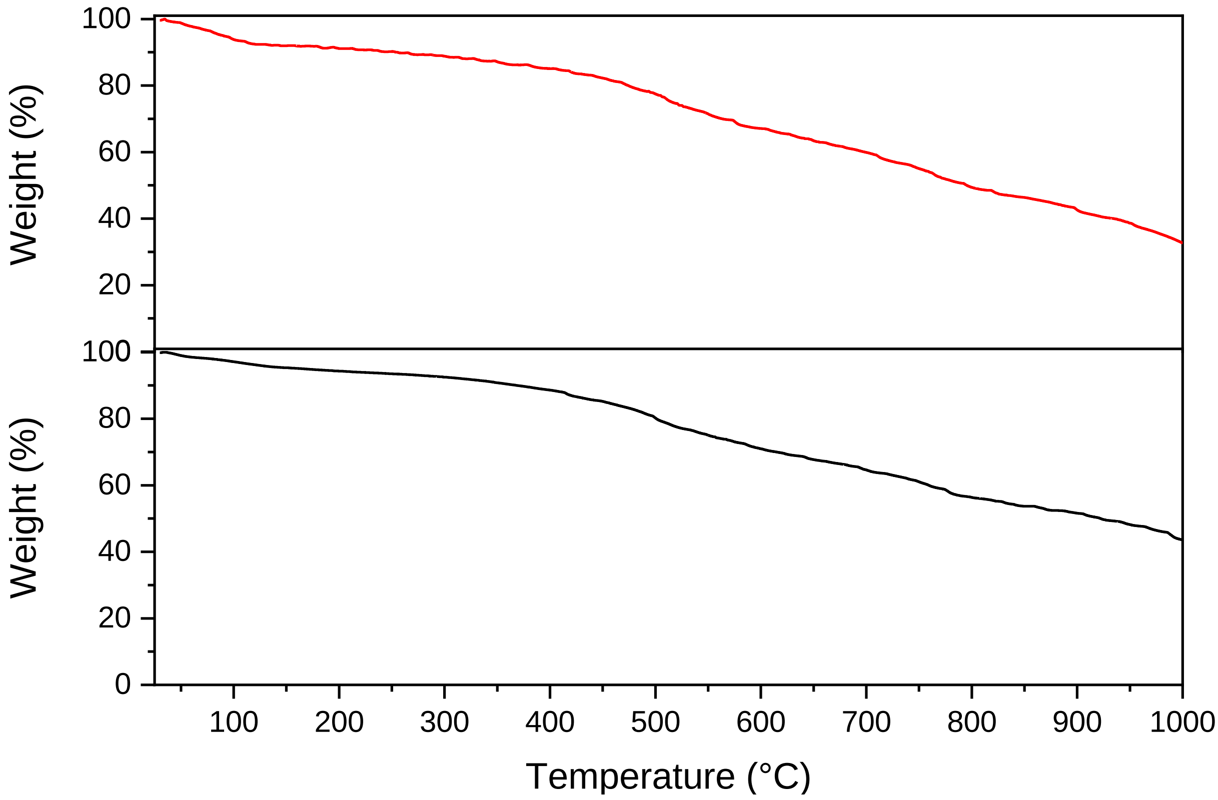


**Figure S13.** TGA analyses of COF PXX$(PhNH_2)_2$-Tp (red line) and COF PXX$(PhNH_2)_2$-TFB (black line).

### 3.5 Scanning electron microscopy (SEM)

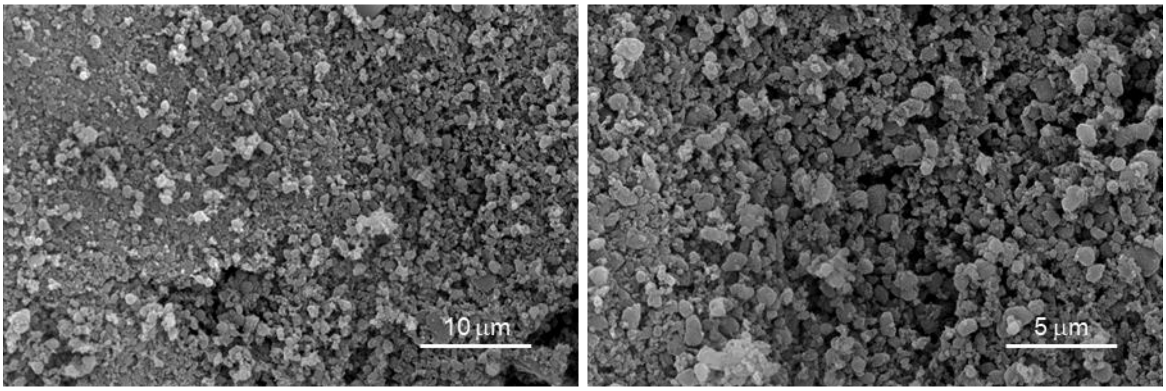


**Figure S14.** SEM images of COF PXX$(PhNH_2)_2$-Tp.

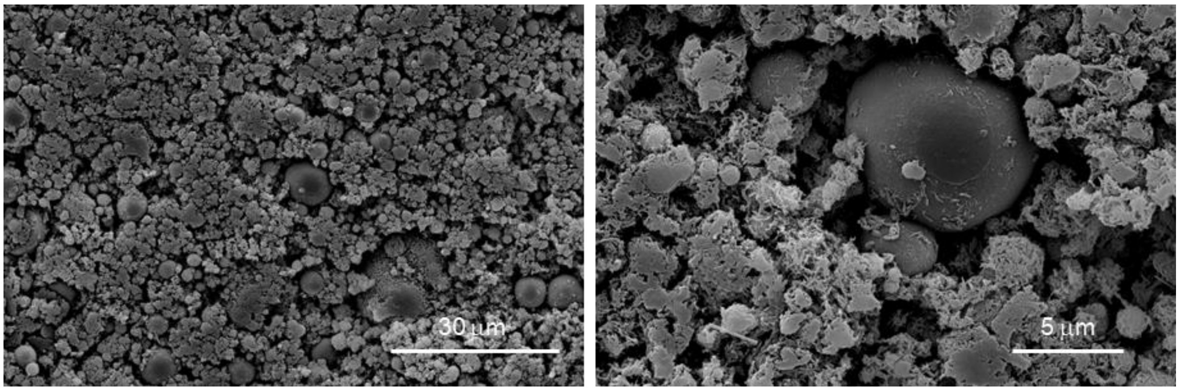


**Figure S15.** SEM images of COF PXX$(PhNH_2)_2$-TFB.

### 3.6 Nitrogen sorption measurements

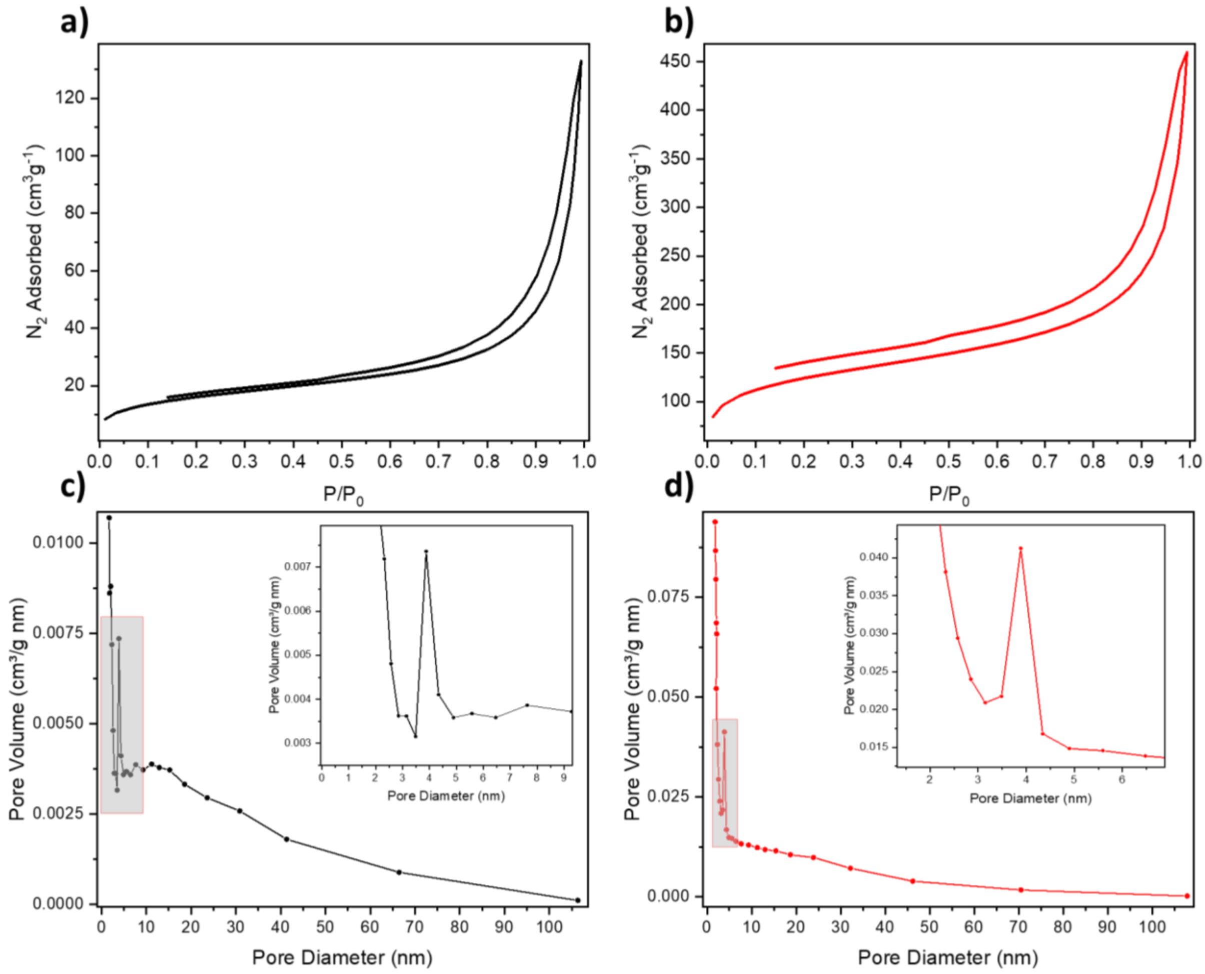


**Figure S16**. a-b) Nitrogen adsorption-desorption isotherms a) COF PXX($(PhNH_2)_2$-TFB and b) COF PXX($(PhNH_2)_2$-Tp; pore size distribution of c) COF PXX($(PhNH_2)_2$-TFB and d) COF PXX($(PhNH_2)_2$-Tp.

**Table S3.** Specific surface area and total pore volume of pores with diameter < 900.732 Å at $P/P_0$=0.98.

| | Specific surface area ($m^2$/g) | Total pore volume ($cm^3$/g) |
|---|---|---|
| COF PXX($(PhNH_2)_2$-TFB | 58 | 0.18 |
| COF PXX($(PhNH_2)_2$-Tp | 422.26 | 0.682 |

# 4 Electrochemical characterization

## 4.1 Three-electrode system

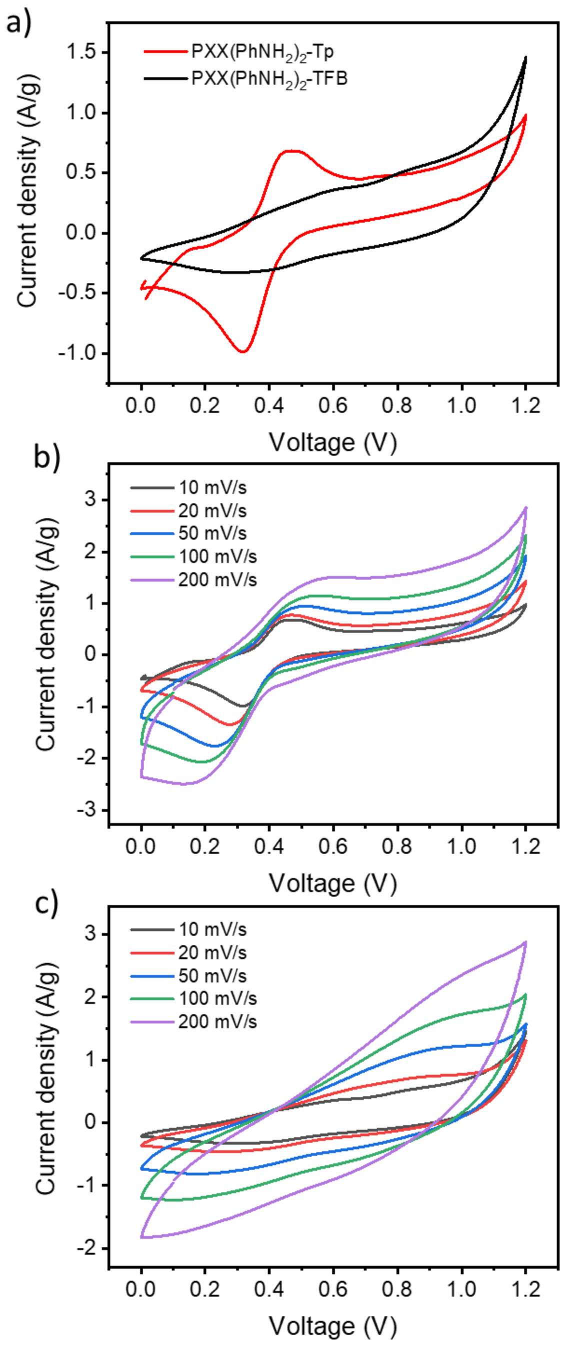


**Figure S17.** a) CV curves of COF PXX$(PhNH_2)_2$-Tp (red curve) and COF PXX$(PhNH_2)_2$-TFB (black curve) at 10 mV/s, b-c) CV curves at different scan rates of b) COF PXX$(PhNH_2)_2$-Tp and c) COF PXX$(PhNH_2)_2$-TFB.

**Table S4.** Physical parameters of PXX$(PhNH_2)_2$-Tp and PXX$(PhNH_2)_2$-TFB.

| Parameter | PXX$(PhNH_2)_2$-Tp | PXX$(PhNH_2)_2$-TFB |
|---|---|---|
| **Surface area ($m^2/g$)** | 422.26 | 58.37 |
| **Pore volume ($cm^3/g$)** | 0.682 | 0.18 |
| **Crystalline** | Low | Medium |
| **Redox activity** | C=O | No |

**4.2 Cyclic voltammetry**

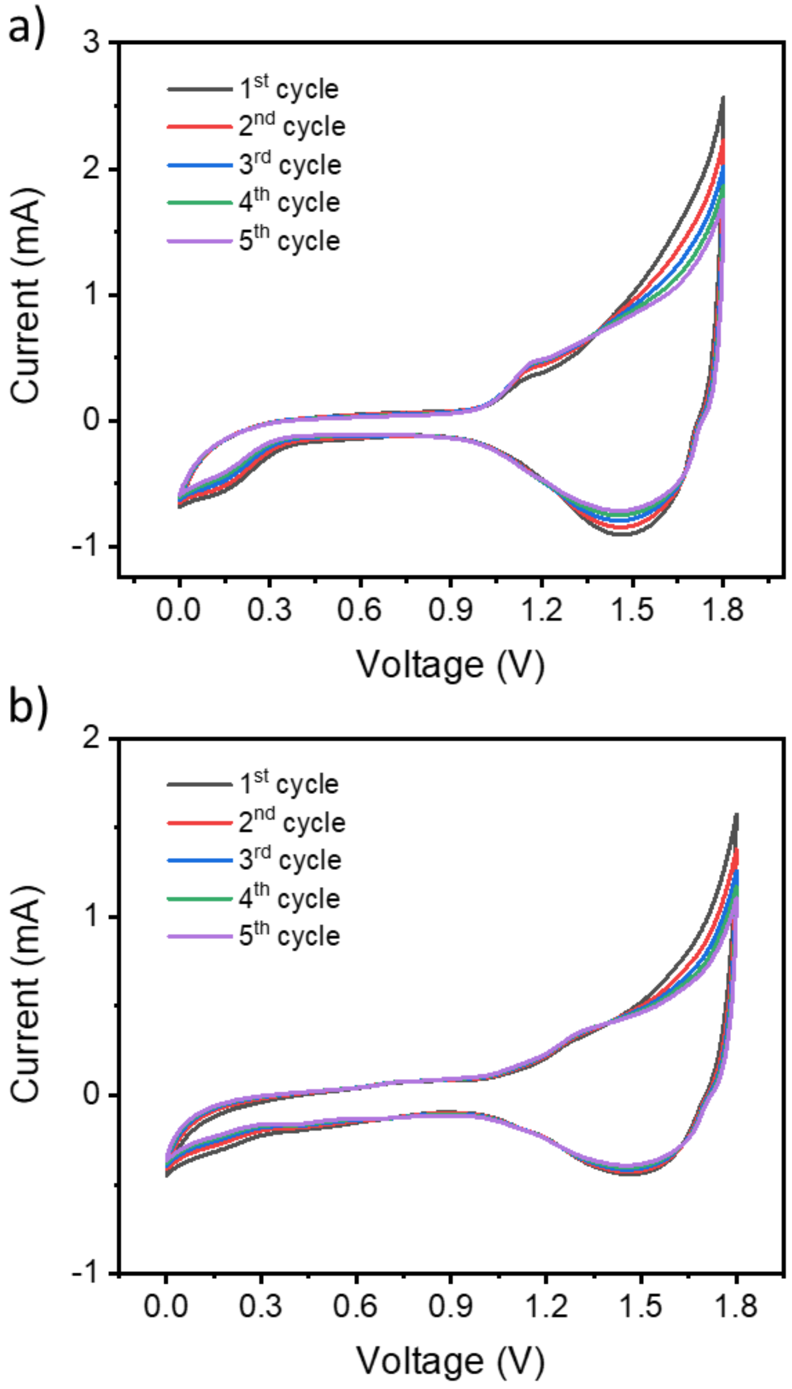


**Figure S185.** CV curves of COF a) PXX(PhNH$_2$)$_2$-Tp and b) PXX(PhNH$_2$)$_2$-TFB during 5 cycles.

### 4.3 Electrochemical Impedance Spectroscopy

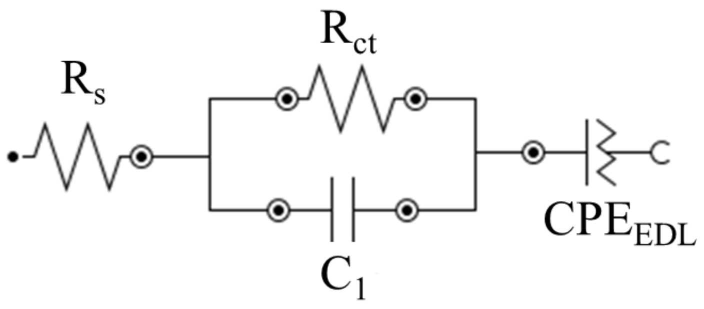


**Figure S19.** The equivalent electric circuit models used for fitting the Nyquist plots. $R_s$: the intrinsic ohmic resistance; $R_{ct}$: charge transfer resistance; $C_1$: capacitance element; $CPE_{EDL}$: constant phase element representing the electrical double layer capacitance (EDLC).

**Table S5.** Fitting parameters obtained from the Nyquist plots

| **Sample** | Rs (Ω) | Rct (Ω) |
|---|---|---|
| PXX(PhNH$_2$)$_2$-Tp | 2.27 | 39.95 |
| PXX(PhNH$_2$)$_2$-TFB | 4.88 | 44.06 |

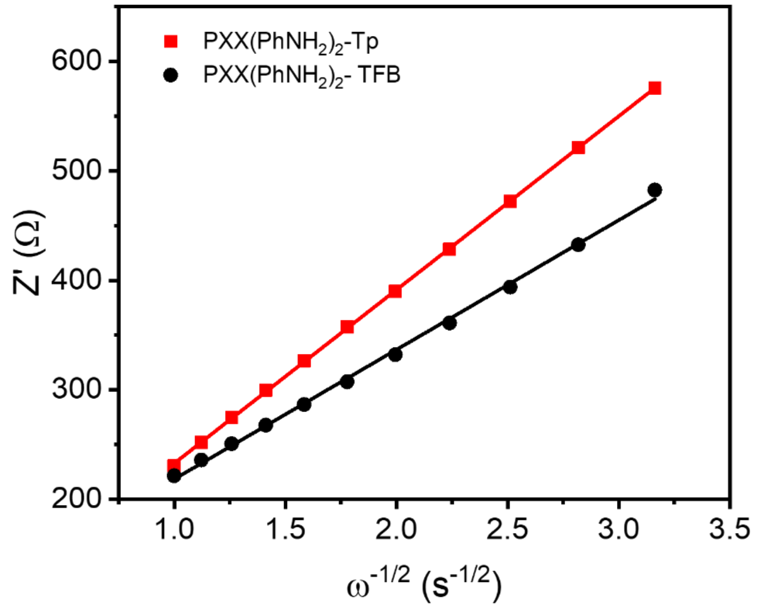


**Figure S20.** Corresponding linear relation between $\omega^{-1/2}$ and Z' in the low frequency region of the Nyquist plots of COF PXX(PhNH$_2$)$_2$-Tp (red curve) and COF PXX(PhNH$_2$)$_2$-TFB (black curve).

**4.4 Galvanostatic charge discharge**

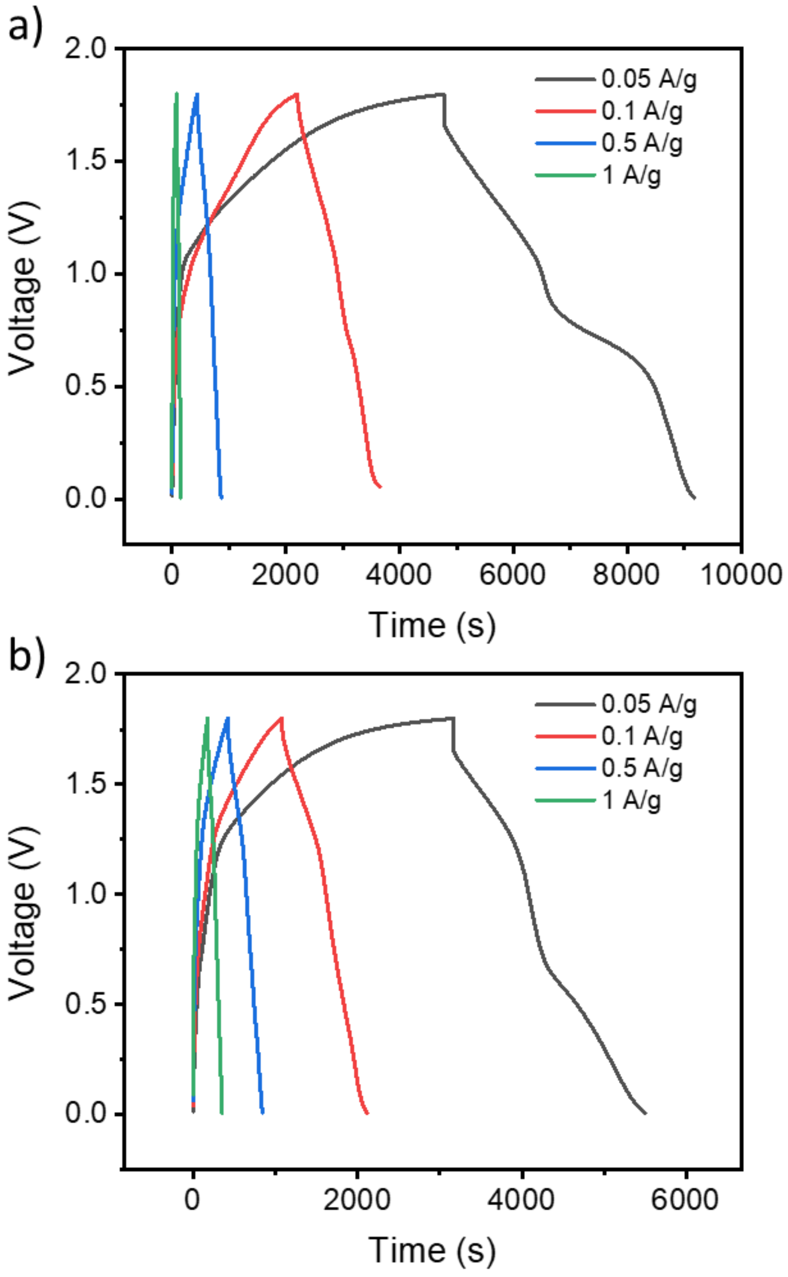


**Figure S21.** GCD profiles of a) COF PXX$(PhNH_2)_2$-Tp and b) COF PXX$(PhNH_2)_2$-TFB at various current densities.

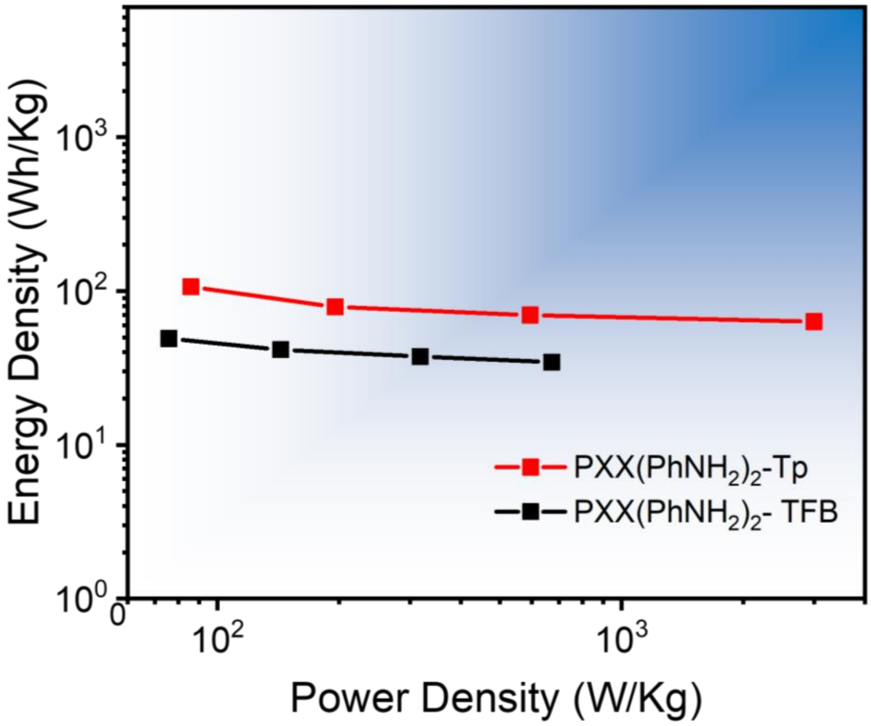


**Figure S6.** Ragone plot for of COF PXX$(PhNH_2)_2$-Tp (red points), and COF PXX$(PhNH_2)_2$-TFB (black points).

**Table S6.** State of the art of the electrochemical performance of various cathode materials in Zn-HSCs.

| Cathode | Anode | Electrolyte | Voltage window (V) | Capacitance/ Capacity | Cyclability | Energy density (Wh/kg) | Power density (kW/kg) | Surface area ($m^2/g$) | Reference |
|---|---|---|---|---|---|---|---|---|---|
| $PXX(PhNH_2)_2$-Tp | Zn foil | 4 M $Zn(CF_3SO_3)_2$ | 0-1.8 | 237 F/g (10 mV/s) | 94% (5000 cycles) | 107 | 3.0 | 422.3 | This work |
| $PXX(PhNH_2)_2$-TFB | Zn foil | 4 M $Zn(CF_3SO_3)_2$ | 0-1.8 | 109 F/g (10 mV/s) | 92% (5000 cycles) | 49 | 0.67 | 58.4 | This work |
| PA-COF | Zn foil | 1 M $ZnSO_4$ | 0.2-1.6 | 247 mAh/g (0.1 A/g) | 99.62 % (10000 cycles) | - | - | 19.6 | [3] |
| $MnO_2$ | PI-COF | 2 M $ZnSO_4$ | 0.9-0 | 92 mAh/g (0.7 A/g) | 85 % (4000 cycles) | 23.9-66.5 | 0.133-4.782 | - | [4] |
| HqTp | Zn foil | 3 M $ZnSO_4$ | 0.2-1.8 | 276 mAh/g (0.125 A/g) | 98 % (1000 cycles) | 240 | 0.109 | 113 | [5] |
| HAQ-COF | Zn foil | 2 M $ZnSO_4$ | 0.2-1.8 | 339 mAh/g (0.1 A/g) | 85 % (10000 cycles) | 75 | 3.262 | 53 | [6] |
| Tp-PTO-COF | Zn foil | 2 M $ZnSO_4$ | 0.2-1.8 | 301.4 mAh/g (0.2 A/g) | 95 % (1000 cycles) | 44-268 | 0.078-4.952 | 601 | [7] |

| | | | | | | | | | |
|---|---|---|---|---|---|---|---|---|---|
| CTFO-NS-700 | Zn foil | 1 M $Zn(CF_3SO_3)_2$ | 0-2 | 296.5 F/g (1 A/g) | 95.7 % (10000 cycles) | 162.5 | 43.8 | 507 | [8] |
| AC | 2D-Zn/Ni | 1 M $ZnSO_4$ | 0.2-1.8 | 468 F/g (0.5 A/g) | 99% (10000 cycles) | 208 | 20 | 2201 | [9] |
| Oxidized carbon nanotubes | Zn foil | 1 M $ZnSO_4$ | 0-1.8 | 53 F/g (0.01 V/s) | 100% (5000 cycles) | - | - | 211 | [10] |
| MOF derived C | Zn foil | 1 M $ZnSO_4$ | 0.1-0.7 | 134 F/g (0.2 A/g) | 99% (10000 cycles) | 36.4 | 0.085 | 85.5 | [11] |
| rGO | Zn foil | 1 M $ZnSO_4$ | 0.2-1.6 | 370.8 F/g (0.1 A/g) | 94.5% (10000 cycles) | 100.9 | 0.07 | - | [12] |
| Porous carbon | Zn foil | 3 M $Zn(CF_3SO_3)_2$ | 0-1.9 | 210 F/g (0.1 A/g) | 93% (80000 cycles) | 106 | 31.4 | 2957 | [13] |
| CNT delaminated $V_2C$ MXene | Zn foil | 1 M $ZnSO_4$ | 0.1-1.1 | 256.6 F/g (1 A/g) | 100% (4000 cycles) | - | - | 61.18 | [14] |
| rGO-MXene | Zn foil | 2 M $ZnSO_4$ | 0.2-1.6 | 129 F/g (0.4 A/g) | 95% (75000 cycles) | 35 | 4 | - | [15] |
| $MnO_2$-CNTs | Mxenes film | 2 M $ZnSO_4$ + 0.1 M $MnSO_4$ | 0.8-1.9 | 115.1 F/g (0.001 V/s) | 83.6% (15000 cycles) | 98.7 | 0.077 | - | [16] |

AC: activated carbon; MOF: metal organic framework; CNT: carbon nanotubes

### 4.5 Post-mortem analysis

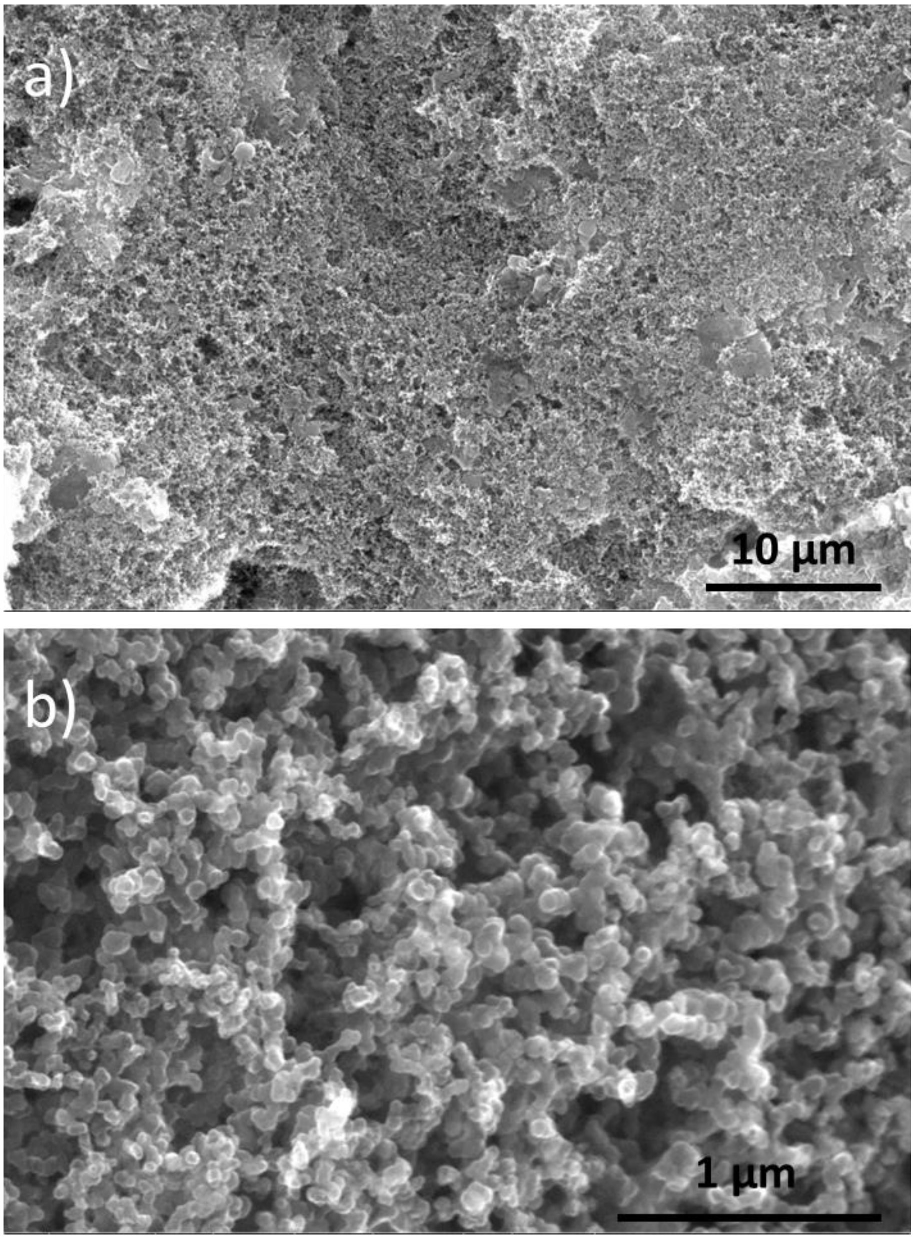


**Figure S23.** SEM images at different magnifications of COF PXX$(PhNH_2)_2$-Tp after 5000 charge/discharge cycles.

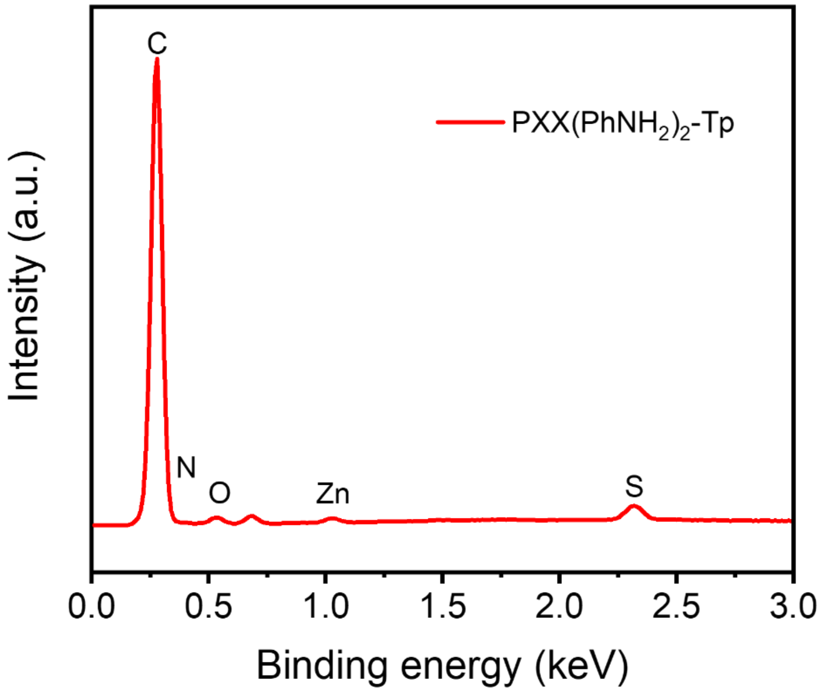


**Figure S247.** EDX spectrum of COF PXX$(PhNH_2)_2$-Tp after 5000 charge/discharge cycles.

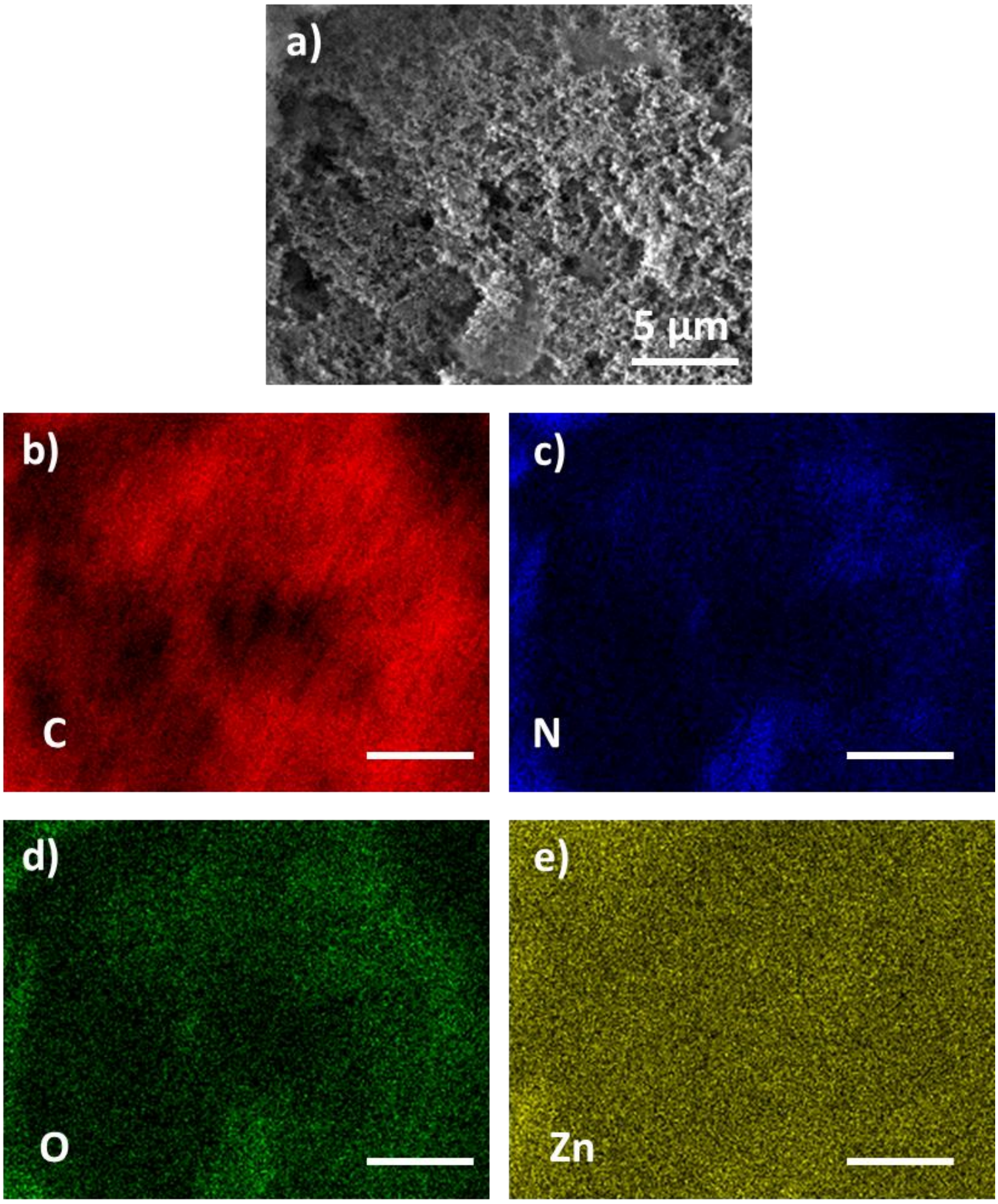


**Figure S25.** EDX mapping analysis of COF PXX$(PhNH_2)_2$-Tp after 5000 charge/discharge cycles. The scale bar is 5 μm in all cases.

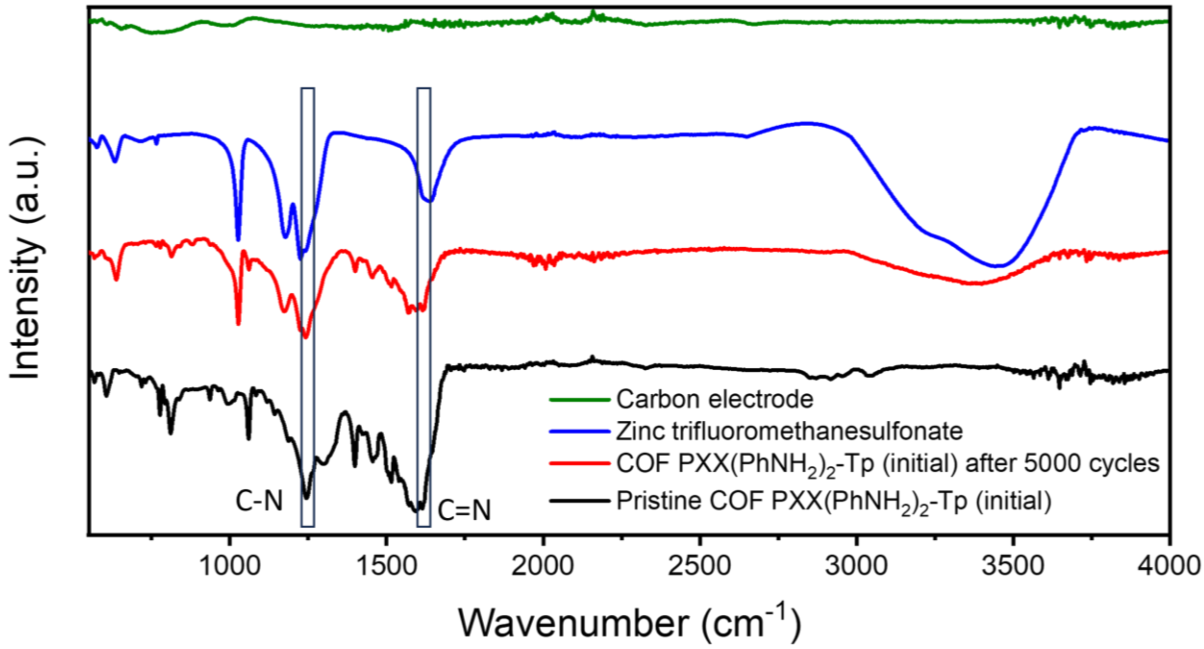


**Figure S26.** FTIR spectra of pristine carbon paper electrode (CPE) (green spectrum), CPE coated with zinc trifluoromethanesulfonate electrolyte (blue spectrum), CPE coated with COF COF PXX($(PhNH_2)_2$-Tp after 5000 charge/discharge cycles (red spectrum) and CPE coated with pristine COF PXX$(PhNH_2)_2$-Tp.

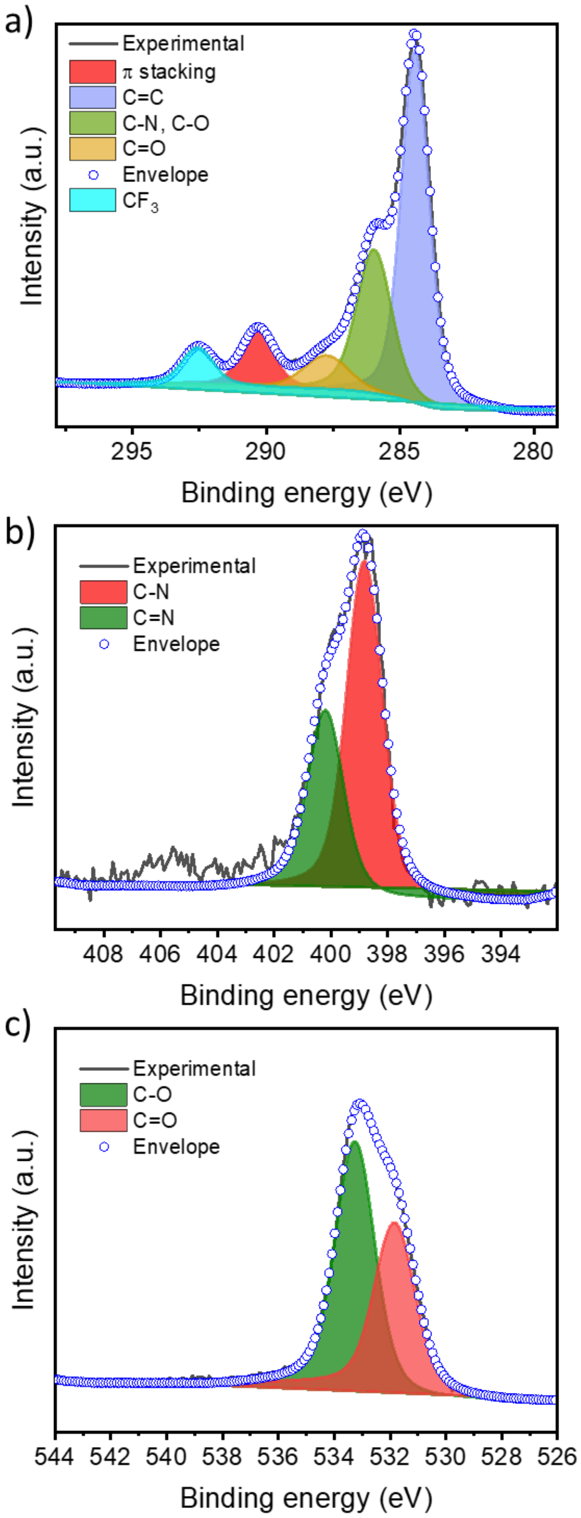


**Figure S27.** a) C1s, b) N1s, c) O1s XPS spectra of COF PXX($PhNH_2$)$_2$-Tp after 5000 charge/discharge cycles.

### 4.6 Charge storage mechanism

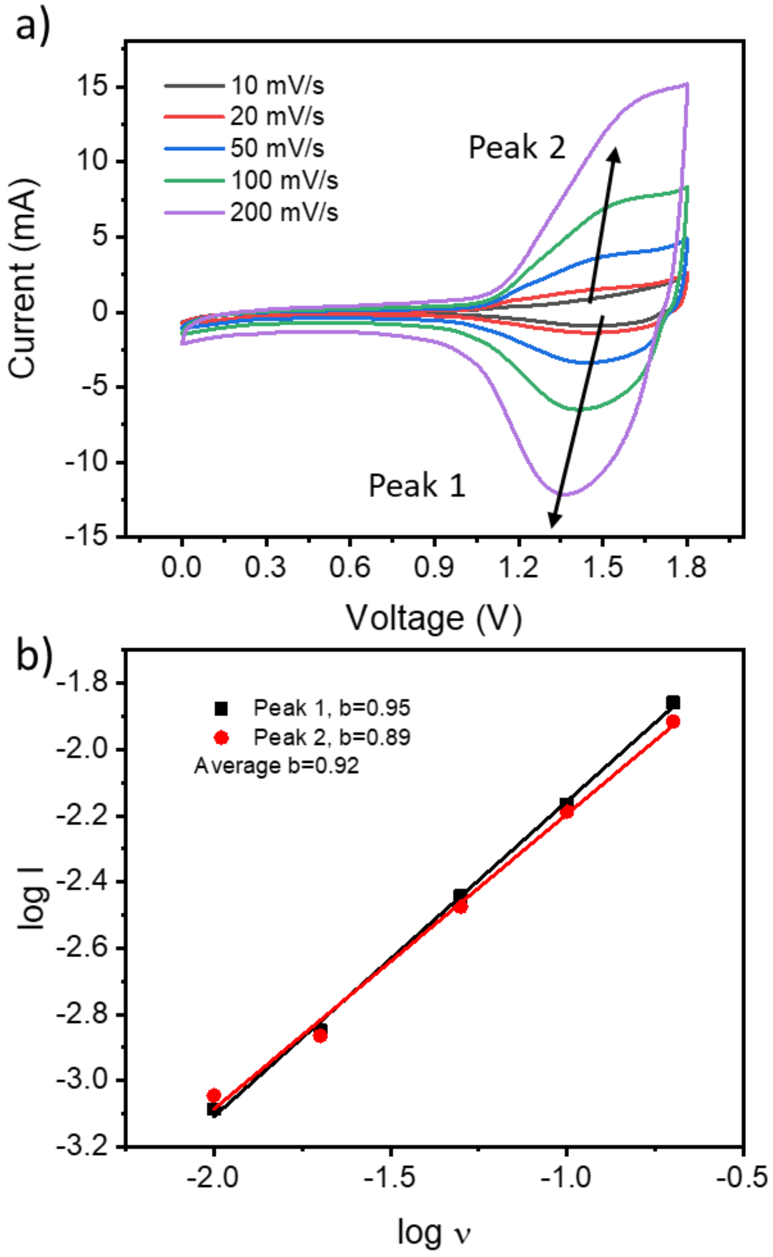


**Figure S288.** a) CV curves of COF PXX($PhNH_2$)$_2$-Tp at various scan rates, b) fitting plots between log(i) and log (υ) at various peak currents.

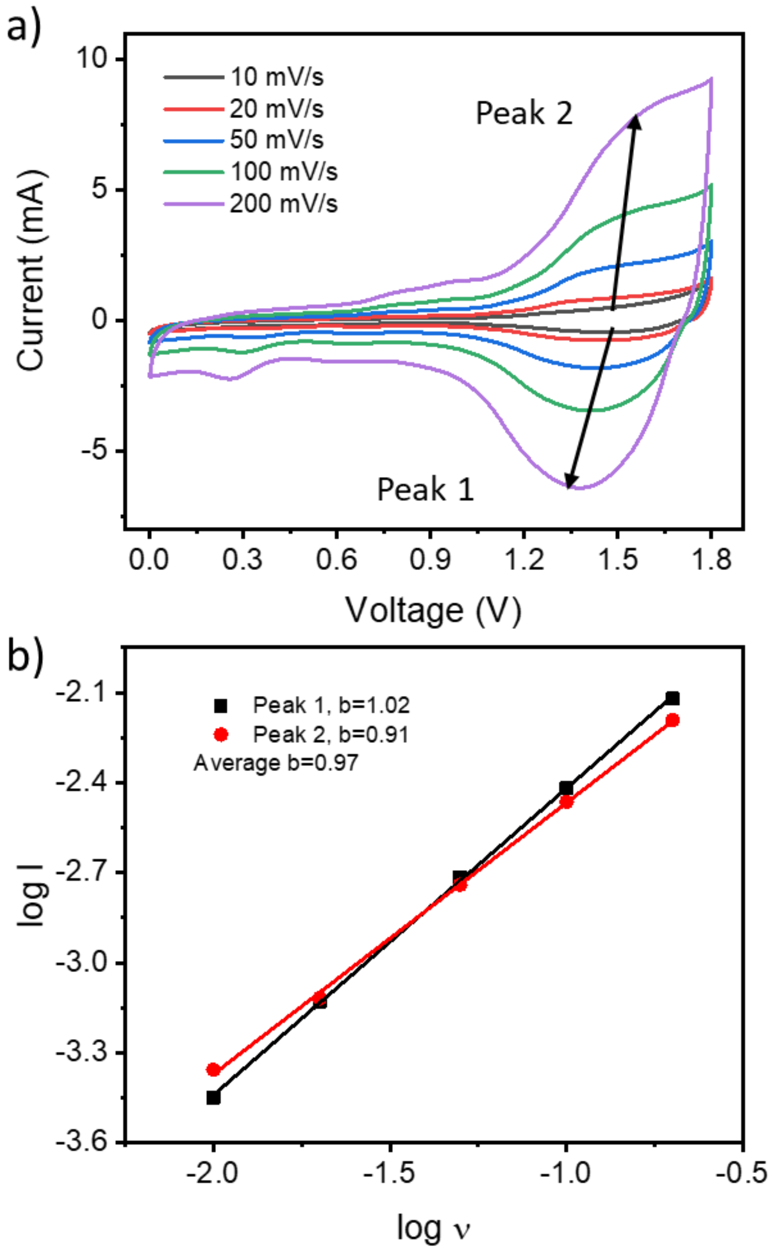


**Figure S29.** a) CV curves of COF PXX($PhNH_2$)$_2$-TFB at various scan rates, b) fitting plots between log(i) and log (υ) at various peak currents.

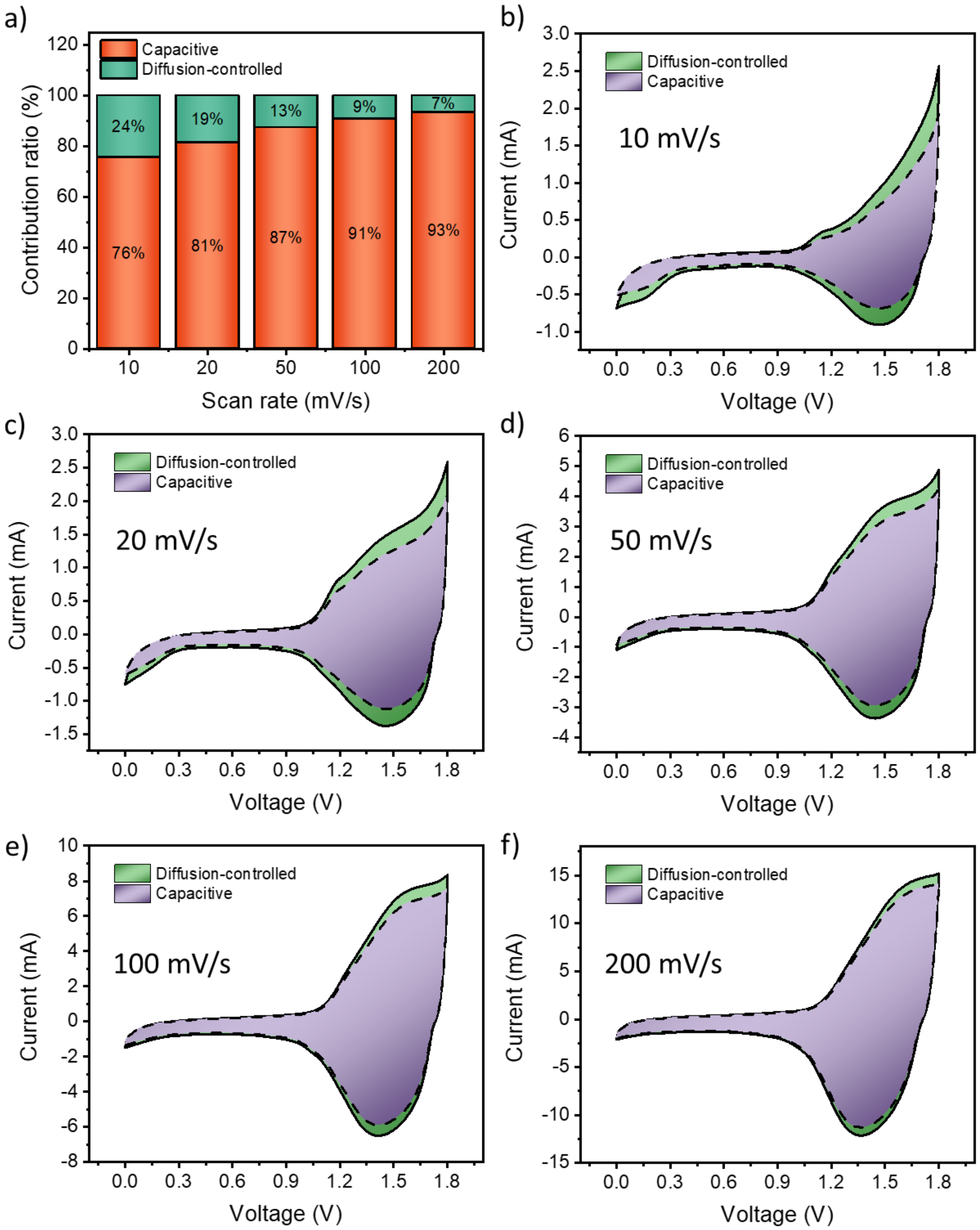


**Figure S30.** a) Capacitive (contribution) and diffusion-controlled contribution of COF PXX$(PhNH_2)_2$-Tp at various scan rates, b-f) capacitive (contribution) and diffusion-controlled contribution fraction for the CV curves of COF PXX$(PhNH_2)_2$-Tp recorded at different scan rates.

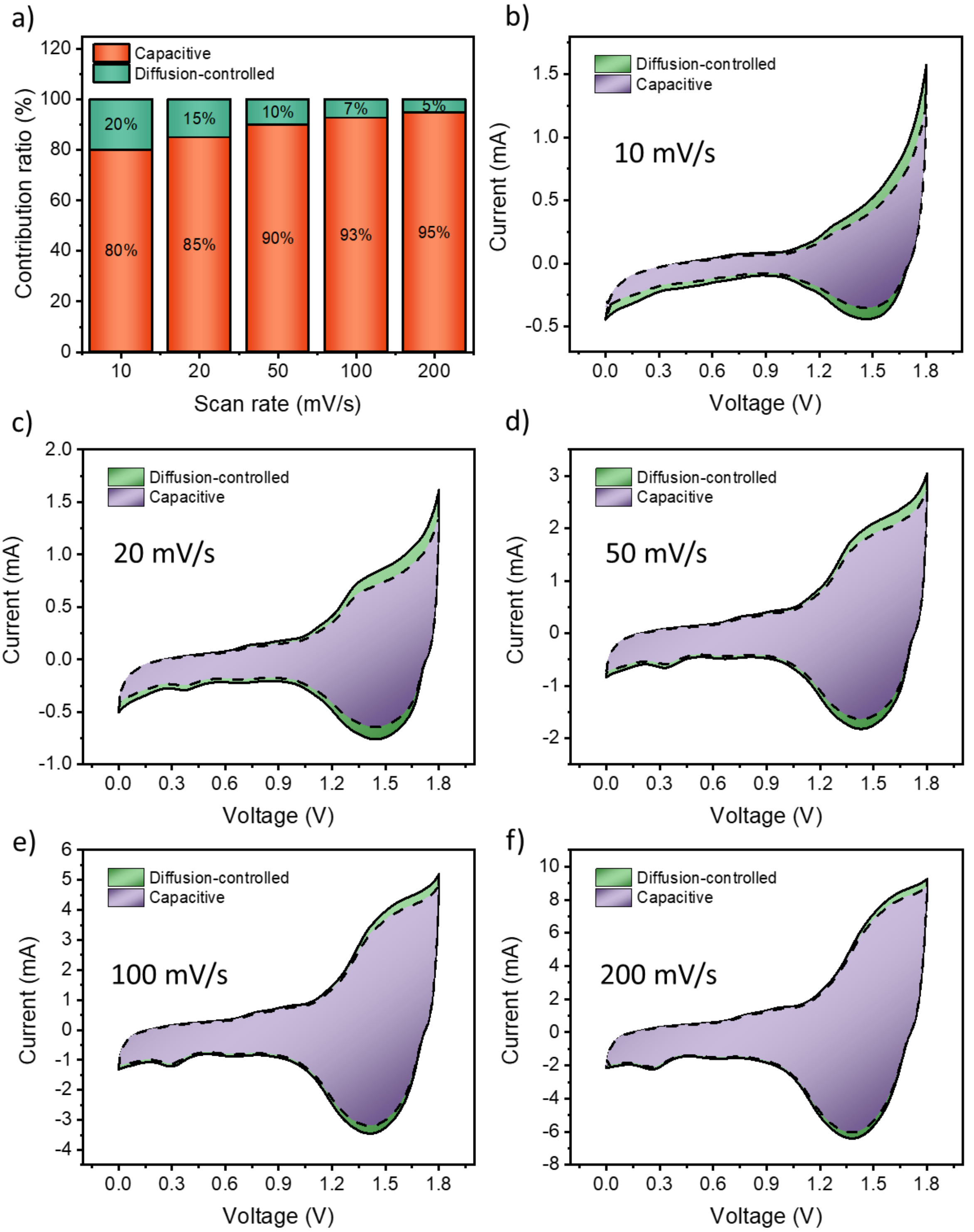


**Figure S31.** a) Capacitive (contribution) and diffusion-controlled contribution of COF PXX$(PhNH_2)_2$-TFB at various scan rates, b-f) capacitive (contribution) and diffusion-controlled contribution fraction for the CV curves of COF PXX$(PhNH_2)_2$-TFB recorded at different scan rates.

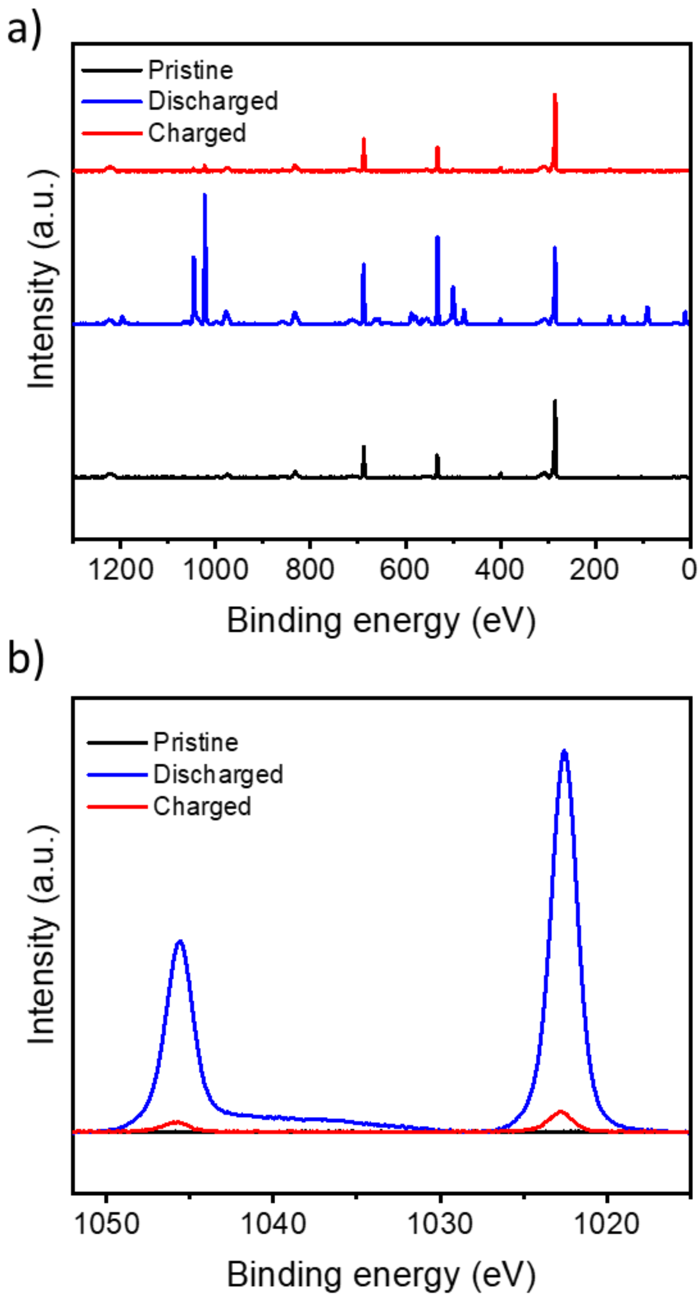


**Figure S32.** a) Survey and b) Zn2p XPS spectra of COF PXX$(PhNH_2)_2$-Tp in pristine, discharged and charged form.

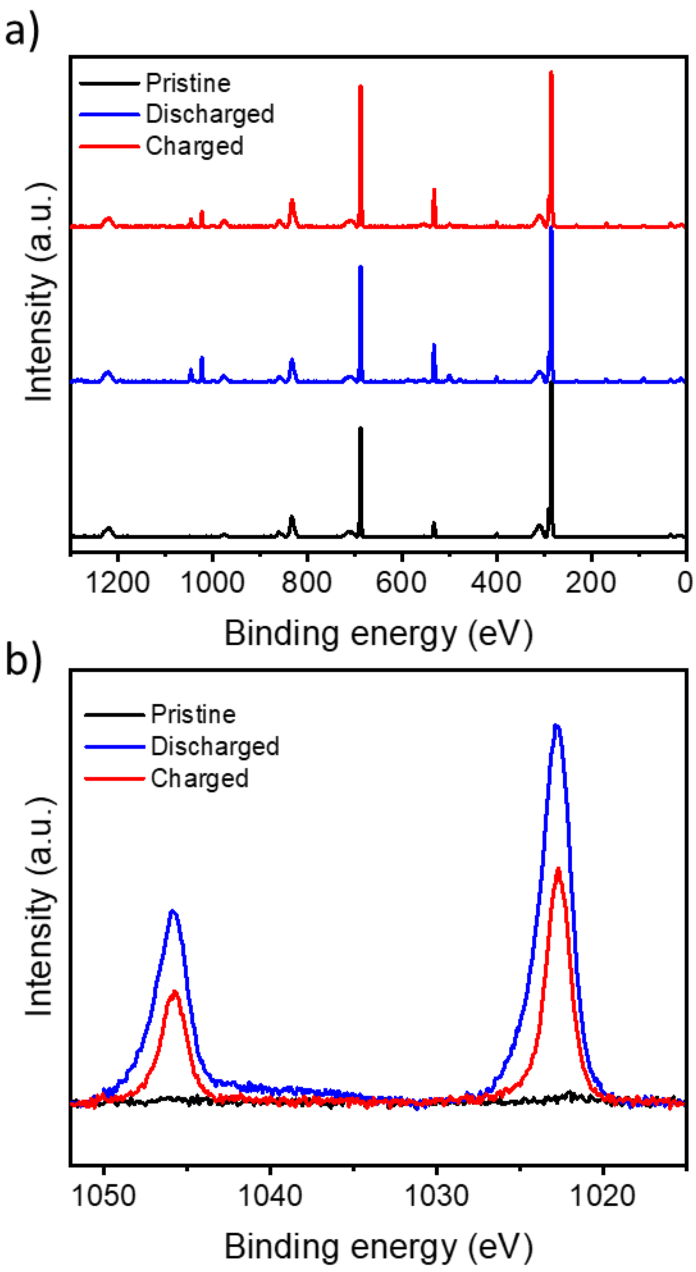


**Figure S339.** a) Survey and b) Zn2p XPS spectra of COF PXX($PhNH_2$)$_2$-TFB in pristine, discharged and charged form.

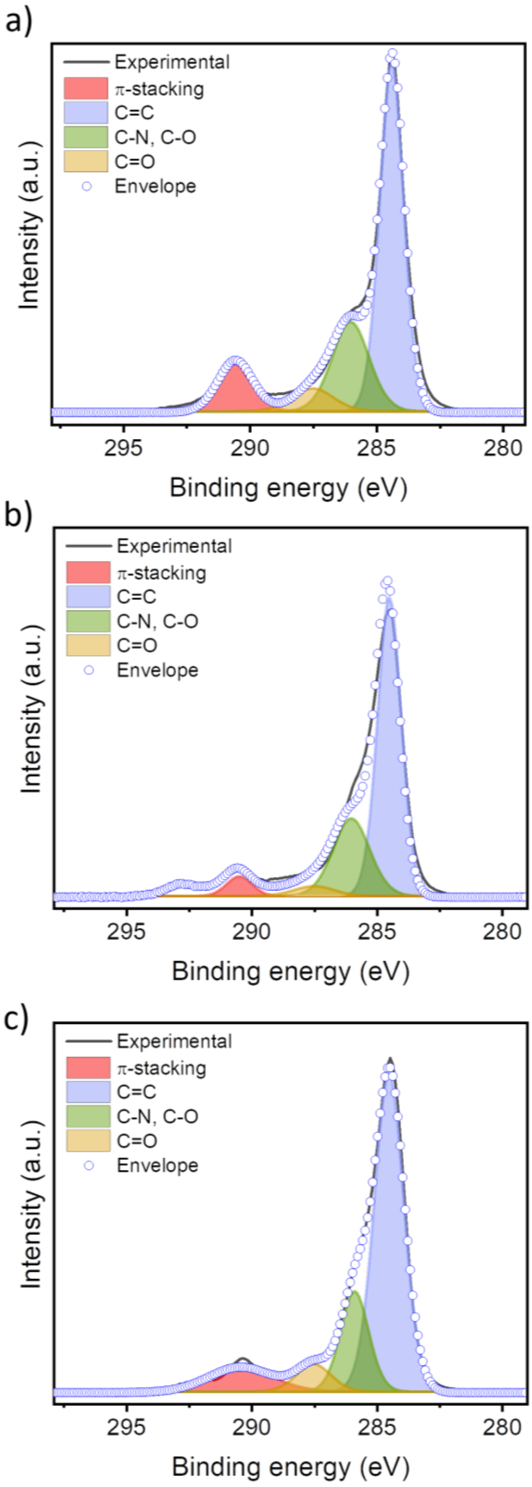


**Figure S3410.** C1S XPS spectra of COF PXX$(PhNH_2)_2$-Tp in a) pristine, b) discharged and c) charged form.

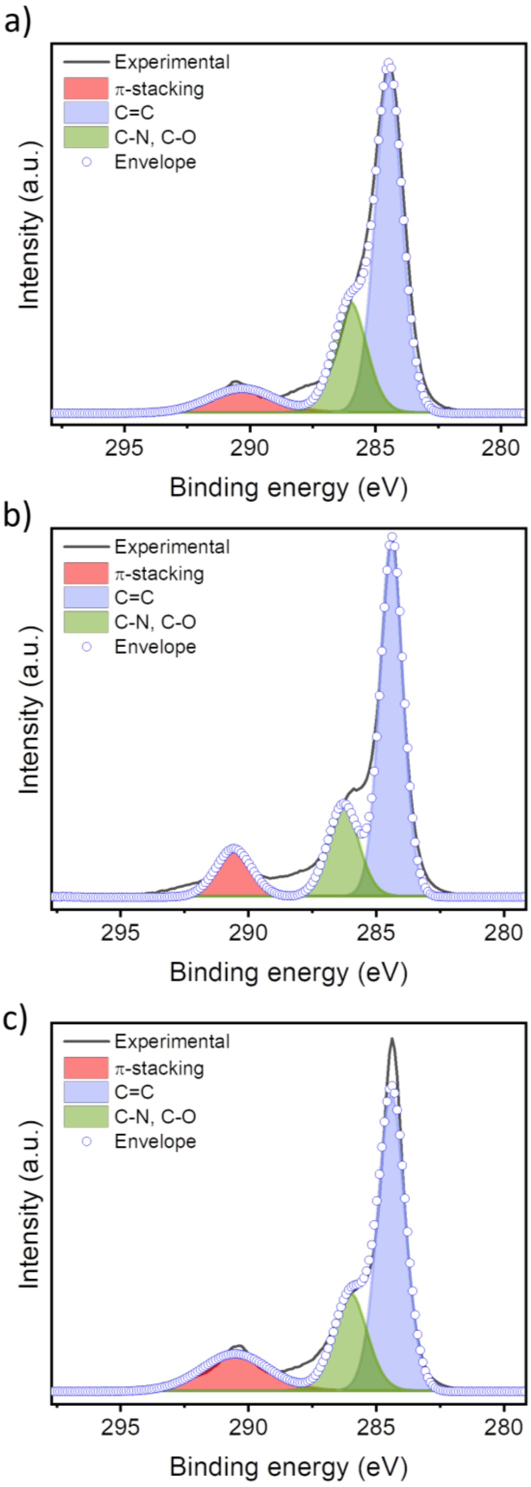


**Figure S35**. C1S XPS spectra of COF PXX($PhNH_2$)$_2$-TFB in a) pristine, b) discharged and c) charged form.

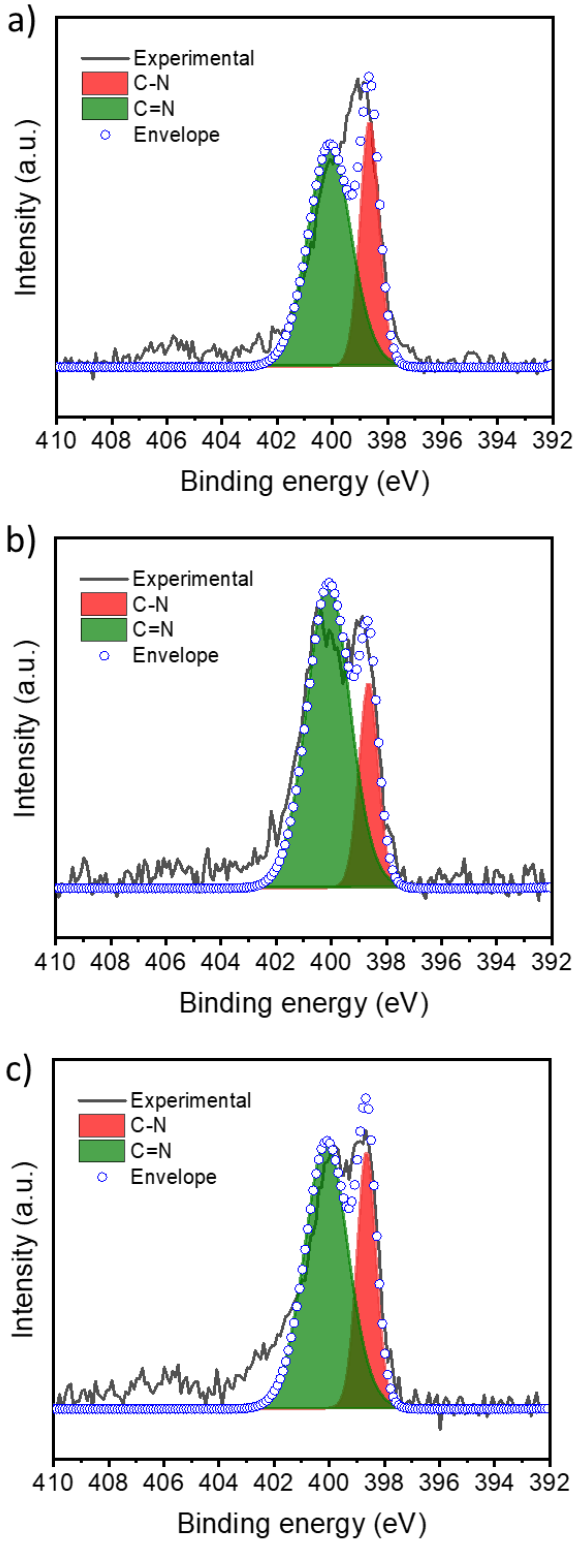


**Figure S36.** N1S XPS spectra of COF PXX$(PhNH_2)_2$-Tp in a) pristine, b) discharged and c) charged form.

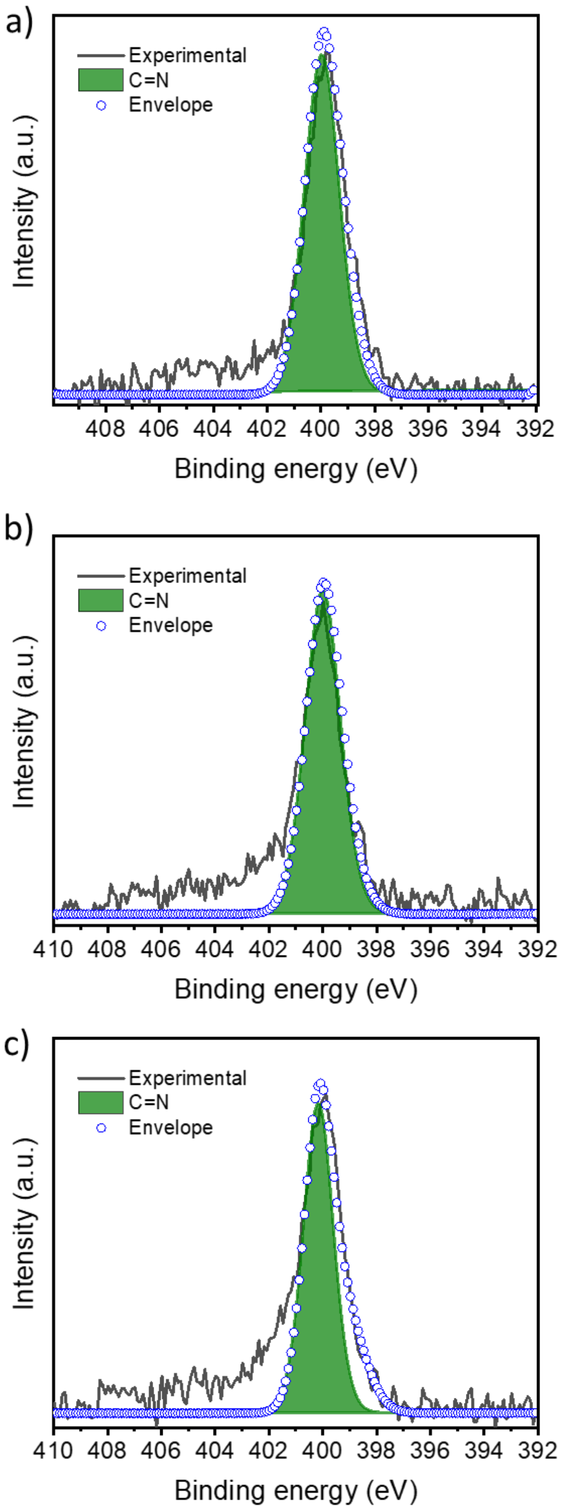


**Figure S37.** N1S XPS spectra of COF PXX$(PhNH_2)_2$-TFB in a) pristine, b) discharged and c) charged form.

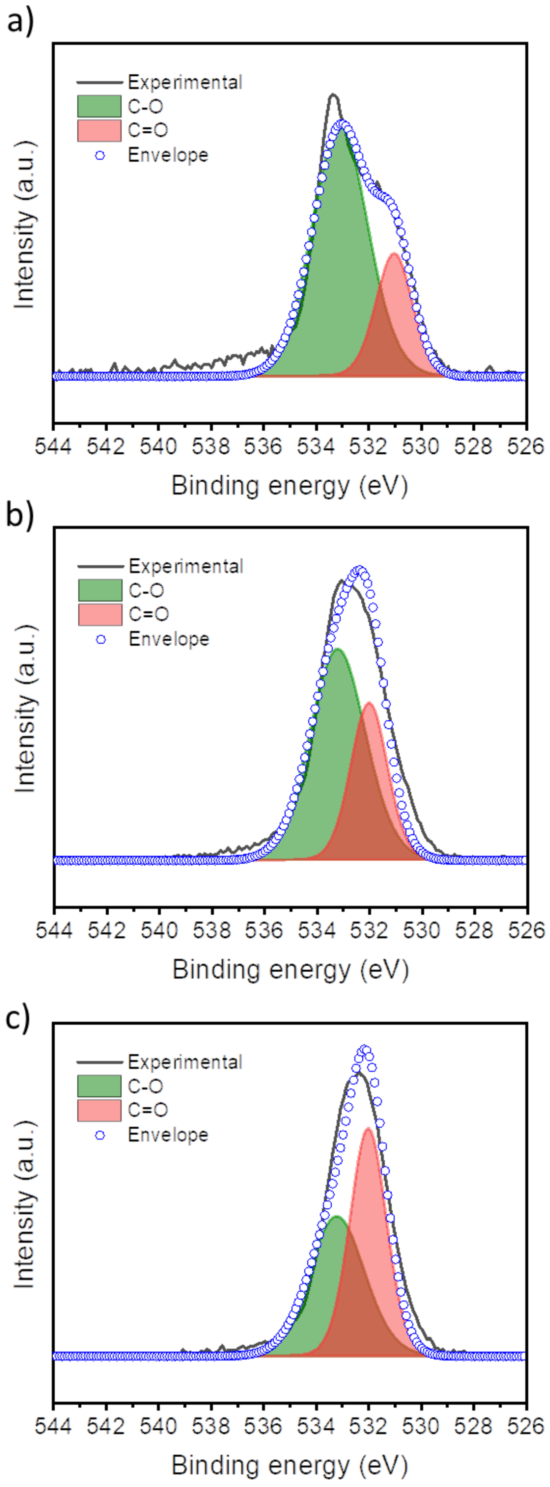


**Figure S38.** O1S XPS spectra of COF PXX$(PhNH_2)_2$-Tp in a) pristine, b) discharged and c) charged form.

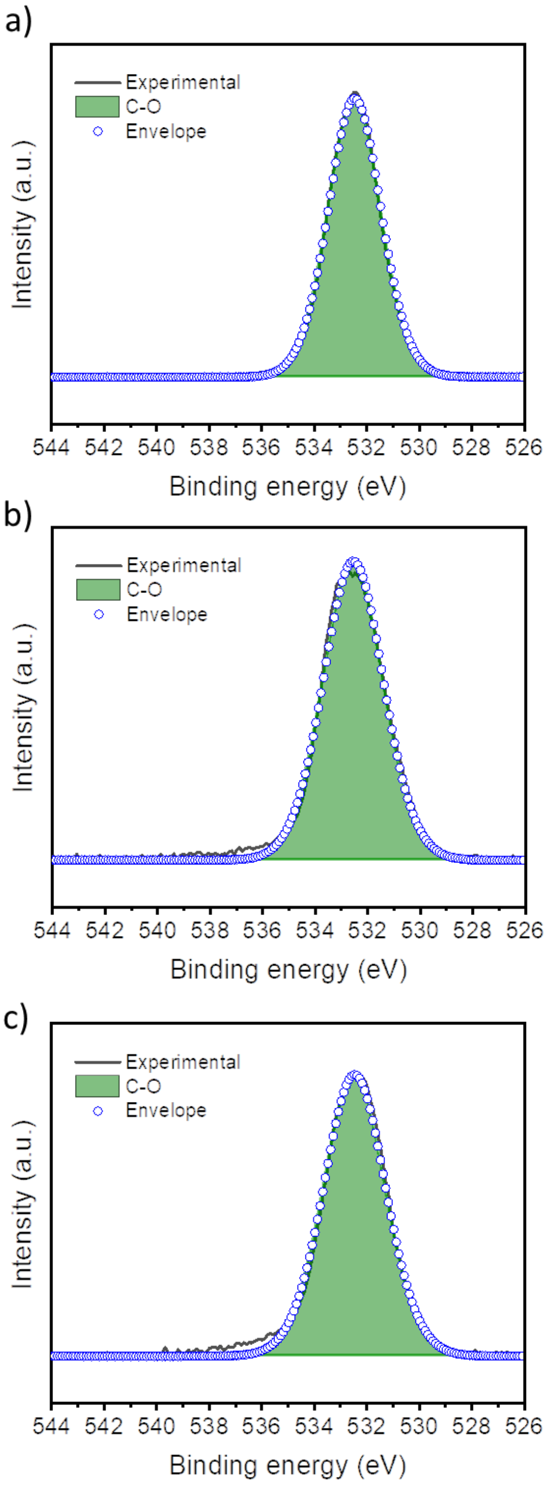


**Figure S39.** O1S XPS spectra of COF PXX($PhNH_2$)$_2$-TFB in a) pristine, b) discharged and c) charged form.

## 5 References


[1] T. Kamei, M. Uryu, T. Shimada, *Organic Letters* **2017**, 19, 2714.

[2] J. Lawrence, G. C. Sosso, L. Đorđević, H. Pinfold, D. Bonifazi, G. Costantini, *Nature Communications* **2020**, 11, 2103.

[3] W. Wang, V. S. Kale, Z. Cao, S. Kandambeth, W. Zhang, J. Ming, P. T. Parvatkar, E. Abou-Hamad, O. Shekhah, L. Cavallo, M. Eddaoudi, H. N. Alshareef, *ACS Energy Lett.* **2020**, 5, 2256.

[4] M. Yu, N. Chandrasekhar, R. K. M. Raghupathy, K. H. Ly, H. Zhang, E. Dmitrieva, C. Liang, X. Lu, T. D. Kühne, H. Mirhosseini, I. M. Weidinger, X. Feng, *J. Am. Chem. Soc.* **2020**, 142, 19570.

[5] A. Khayum M, M. Ghosh, V. Vijayakumar, A. Halder, M. Nurhuda, S. Kumar, M. Addicoat, S. Kurungot, R. Banerjee, *Chem. Sci.* **2019**, 10, 8889.

[6] W. Wang, V. S. Kale, Z. Cao, Y. Lei, S. Kandambeth, G. Zou, Y. Zhu, E. Abouhamad, O. Shekhah, L. Cavallo, M. Eddaoudi, H. N. Alshareef, *Adv. Mater.* **2021**, 33, 2103617.

[7] D. Ma, H. Zhao, F. Cao, H. Zhao, J. Li, L. Wang, K. Liu, *Chem. Sci.* **2022**, 13, 2385.

[8] B. Liu, T. Quan, M. Yang, Y. Liu, H. Chen, H. Li, *Chem Eng J* **2023**, 461, 141925.

[9] G.-H. An, J. Hong, S. Pak, Y. Cho, S. Lee, B. Hou, S. Cha, *Adv. Energy Mater.* **2020**, 10, 1902981.

[10] Y. Tian, R. Amal, D.-W. Wang, *Front. Energy Res.* **2016**, 4.

[11] T. Xiong, Y. Shen, W. S. V. Lee, J. Xue, *Nano Mater. Sci.* **2020**, 2, 159.

[12] G. Sun, Y. Xiao, B. Lu, X. Jin, H. Yang, C. Dai, X. Zhang, Y. Zhao, L. Qu, *ACS Appl. Mater. Interfaces* **2020**, 12, 7239.

[13] S. Wu, Y. Chen, T. Jiao, J. Zhou, J. Cheng, B. Liu, S. Yang, K. Zhang, W. Zhang, *Adv. Energy Mater.* **2019**, 9, 1902915.

[14] C. Wang, S. Wei, S. Chen, D. Cao, L. Song, *Small Methods* **2019**, 3, 1900495.

[15] Q. Wang, S. Wang, X. Guo, L. Ruan, N. Wei, Y. Ma, J. Li, M. Wang, W. Li, W. Zeng, *Adv. Electron. Mater.* **2019**, 5, 1900537.

[16] S. Wang, Q. Wang, W. Zeng, M. Wang, L. Ruan, Y. Ma, *Nanomicro Lett.* **2019**, 11, 70.
